# Functional Genetic Diversity and Culturability of Petroleum-Degrading Bacteria Isolated From Oil-Contaminated Soils

**DOI:** 10.3389/fmicb.2018.01332

**Published:** 2018-06-20

**Authors:** Ji-Quan Sun, Lian Xu, Xue-Ying Liu, Gui-Fang Zhao, Hua Cai, Yong Nie, Xiao-Lei Wu

**Affiliations:** ^1^Department of Energy & Resources Engineering, College of Engineering, Peking University, Beijing, China; ^2^School of Environment, Tsinghua University, Beijing, China

**Keywords:** ambient temperature, bacteria, bioremediation, culturability, petroleum degradation

## Abstract

In this study, we compared the culturability of aerobic bacteria isolated from long-term oil-contaminated soils via enrichment and direct-plating methods; bacteria were cultured at 30°C or ambient temperatures. Two soil samples were collected from two sites in the Shengli oilfield located in Dongying, China. One sample (S0) was close to the outlet of an oil-production water treatment plant, and the other sample (S1) was located 500 m downstream of the outlet. In total, 595 bacterial isolates belonging to 56 genera were isolated, distributed in Actinobacteria, Firmicutes, Bacterioidetes, and Proteobacteria. It was interesting that Actinobacteria and Firmicutes were not detected from the 16S rRNA gene clone library. The results suggested the activation of rare species during culture. Using the enrichment method, 239 isolates (31 genera) and 96 (22 genera) isolates were obtained at ambient temperatures and 30°C, respectively, from S0 soil. Using the direct-plating method, 97 isolates (15 genera) and 163 isolates (20 genera) were obtained at ambient temperatures and 30°C, respectively, from two soils. Of the 595 isolates, 244 isolates (41.7% of total isolates) could degrade *n*-hexadecane. A greater number of alkane-degraders was isolated at ambient temperatures using the enrichment method, suggesting that this method could significantly improve bacterial culturability. Interestingly, the proportion of alkane degrading isolates was lower in the isolates obtained using enrichment method than that obtained using direct-plating methods. Considering the greater species diversity of isolates obtained via the enrichment method, this technique could be used to increase the diversity of the microbial consortia. Furthermore, phenol hydroxylase genes (*pheN*), medium-chain alkane monooxygenases genes (*alkB* and *CYP153A*), and long-chain alkane monooxygenase gene (*almA)* were detected in 60 isolates (11 genotypes), 91 isolates (27 genotypes) and 93 isolates (24 genotypes), and 34 isolates (14 genotypes), respectively. This study could provide new insights into microbial resources from oil fields or other environments, and this information will be beneficial for bioremediation of petroleum contamination and for other industrial applications.

## Introduction

Microbes not only have crucial roles in global carbon and nitrogen cycles, but also play an important role in industrial processes, such as bioremediation. Culture-independent methods can give us detailed information regarding the diversity of microbial communities across the world, and they can help to define functional genes via metagenomic, metatranscriptomic, and metaproteomic approaches. However, it is still important to study the functions and ecological roles of these microbes in their ecosystems; these studies are usually performed using pure cultures. Currently, the majority of microbes remain uncultivated (Hobbie et al., 1977; Roszak and Colwell, 1987). Therefore, improvements in the culturability of microbes are becoming increasingly important for understanding their functions in the environments, and their potential applications.

Bacterial alkane and aromatic compounds degradation are essential for bioremediation of petroleum-contaminated environments. More than 60 genera of aerobic bacteria and 5 genera of anaerobic bacteria that can degrade *n*-alkanes and more than 100 microbial genera than can degrade aromatic compounds have been reported. Alkane hydroxylases (AHs), which catalyze the hydroxylation of alkanes, are key enzymes in aerobic degradation of alkanes by bacteria. The integral-membrane alkane monooxygenase (AlkB)-related AHs and the cytochrome P450 CYP153 family are two common AHs in bacteria (Nie et al., 2014a). Furthermore, flavin-binding LC-alkane monooxygenase (*almA*) and thermophilic soluble LC-alkane monooxygenase (*ladA*) genes have also been found to be involved in hydroxylation of alkanes (Wentzel et al., 2007; Wang and Shao, 2012). Phenol hydroxylation is a vital step in biodegradation of phenolics, and is catalyzed by phenol hydroxylase. Among the phenol hydroxylase systems, two main classes have been reported, the “two-gene-component” and the “six-gene-component” systems (Powlowski and Shingler, 1994).

However, compared to known isolates, there are still many more unknown petroleum-degrading bacteria in the environment (Nie et al., 2014a). Culture-independent approaches using both, 16S rRNA genes (Jiao et al., 2016) and functional genes (Yang et al., 2014), have been used for isolation of diverse bacteria from petroleum contaminated soils. There are more studies focusing on the succession of bacterial community structures in oil-contaminated soils during bioremediation (Ai-Kindi and Abed, 2016; Leewis et al., 2016; Pacwa-Plociniczak et al., 2016; Wu et al., 2016, 2017). It has been shown that bacterial compositions differ significantly between culture-dependent and -independent methods (Stefani et al., 2015). After years of enrichment, the diverse microbes in petroleum contaminated soils provide an ideal species pool for isolating petroleum-degrading bacteria (Bhattacharya et al., 2003; M'rassi et al., 2015; El Mahdi et al., 2016). However, compared to the microbial diversity in petroleum contaminated soils estimated using culture-independent studies, the number of isolates obtained from these environments is still limited and the functions of majority of the microbes in such environments are still unknown.

The limitations of culturing such microbes may be due to failure to mimic essential aspects of bacterial environments, such as nutrition, environmental conditions, and microbial interactions (Zengler et al., 2002). Previous studies have tried to improve the culturability of microbes isolated from the environment by varying incubating conditions, such as using different medium (Gartner et al., 2011; Sun et al., 2014b), temperature (Weichart et al., 1992; Junge et al., 2002), pressure (Gartner et al., 2011), as well as adding antioxidants (Muresu et al., 2013). Few studies have focused on the effects of oscillations in ambient-temperature on bacterial culturability. Interestingly, bacterial strains also synchronize their circadian rhythms to day/night cycles (Huang et al., 1990), and oscillations in ambient-temperature in nature may influence the growth of microbes. In this study in order to investigate the functions of microbes in petroleum-contaminated soils, we improved the culturability of microbes by simulating ambient temperatures in nature, and characterized new petroleum-degrading bacteria from two different petroleum contaminated soils. The distribution of genes associated with petroleum degradation was also investigated.

## Materials and methods

### Sample collection

Two soil samples were collected from two sites in the Shengli oilfield located in Dongying, Shandong Province, China. One sample (S0) was close to the outlet of an oil-production water treatment plant, and the other sample (S1) was located 500 m downstream of the outlet. The soil at these sites had been contaminated by crude oil for more than 10 years. Soil samples were transported to the laboratory at 4°C immediately following collection. The characteristics of the soil, analyzed by standard methods, are listed in Table S1.

The oil-production water used for cultivating the bacterial strains was sampled from the Gudao wastewater treatment plant in the same oil field. The physical-chemical properties of the oil-production water were determined using standard methods. The characteristics of the oil-production water were as follows: pH 8.5, 22.82 mg total nitrogen l^−1^, 0.18 mg total phosphorous l^−1^, 1,640 mg COD_Cr_ l^−1^. The heavy crude oil used as carbon and energy source was collected directly from the outlet of the production well.

### 16S rRNA gene clone library analysis

Extraction of DNA from oil-contaminated soil and amplification of 16S rRNA genes were carried out as previously described (Yu et al., 2011). PCR products were purified using the PCR-product purification kit (BioTech, China), and were ligated into pGEM T-easy vectors (Promega, USA) according to manufacturer's instruction. The ligated products were transformed into *Escherichia coli* DH5α, and ampicillin resistant colonies were picked up. The inserted fragments from each colony were amplified using the primers SP6 (ATT TAG GTG ACA CTA TAG) and T7 (TAA TAC GAC TCA CTA TAG GG). Amplified products were separated into different types using restriction fragment length polymorphism (RFLP) analysis with *Rsa*I and *Hha*I in order to pick up clones with different 16S rRNA gene insertion. In total, 55 positive clones were picked up from the clone library; these clones were subdivided into 21 different types according to their RFLP results. Thirty clones were selected for DNA sequencing.

### Bacteria isolation

Aerobic bacteria were isolated from soil sample S0 using two different strategies. In the first method, soil sample S0 was immediately transferred to the appropriate media for enrichment after being transported to the laboratory. Three kinds of media were used for enrichment. The G3 mineral salt medium consisted of 5.0 g NaCl l^−1^, 1.0 g K_2_HPO_4_ l^−1^, 1.0 g NH_4_H_2_PO_4_ l^−1^, 1.0 g NH_4_SO_4_ l^−1^, 0.2 g MgSO_4_·7H_2_O l^−1^, and 3.0 g KNO_3_ l^−1^ in ddH_2_O, adjusted to pH 8.0–8.5. The G2 medium was made using oil-production water supplemented with 1.0 g yeast extract l^−1^. The G5 mineral salt medium had the same mineral salt concentration and pH as the G3 medium, but the salts were dissolved in 0.9 l oil-production water and 0.1 l ddH_2_O. One gram of sampled soil was added into each medium supplemented with 1% sterilized heavy crude oil. Cultures were then incubated at ambient, or at a constant temperature of 30°C; cultures were shaken at 150 rpm. Cultures were passaged using fresh media supplemented with 2–10% crude oil every 20 days. After three passages, the enriched cultures were serially diluted and plated onto agars. Agar plates were incubated at the same temperatures as the enrichment temperature. After a 14–day incubation period, all colonies on agar plates were collected for further investigation.

For the second strategy, bacteria were directly isolated from soil samples without enrichment. Soil samples were diluted with the 10-fold dilution method, and the dilutions were plated on oil-production water agar (PW agar: 1000 ml of oil-production water, 15 g of agar) and mineral salt agar (MSM agar: 0.5 g NaCl l^−1^, 1 g NH_4_H_2_PO_4_ l^−1^, 1 g (NH_4_)_2_SO_4_ l^−1^, 0.2 g MgSO_4_·7H_2_O l^−1^, 3 g KNO_3_ l^−1^, 1 g K_2_HPO_4_ l^−1^, 15 g agar l^−1^). The agar plates were incubated at either ambient or constant temperature of 30°C in the dark. After a 1-week incubation period, all colonies present on the plates were collected for further investigation. Bacteria were also directly isolated from soil samples using the same method.

The prefixes of the isolate names were proposed according to sampling sites, the isolation medium, and the isolation temperature (Table S2). Purified isolates were primarily identified via 16S rRNA gene analysis, which was performed according to previously described protocol (Wang et al., 2007b). The 16S rRNA gene sequence obtained was analyzed using the BLAST tool in NCBI (http://blast.ncbi.nlm.nih.gov/Blast.cgi) and Classifier in RDP (http://rdp.cme.msu.edu/classifier/classifier.jsp). Reference sequences were retrieved from GenBank. After multiple sequence alignments using the CLUSTAL_X tool and manual correction, a phylogenetic tree based on the 16S rRNA gene was constructed using the neighbor-joining method (Saitou and Nei, 1987) contained in the MEGA 6.0 software (Tamura et al., 2013). Tree topology was evaluated with bootstrap analysis based on 1,000 resampling replicates.

### Amplification of *alkB, CYP153A, alma*, and *pheN* genes

Genomic DNA was extracted from the isolates as previously described (Wang et al., 2007b). The phenol hydroxylase genes *pheN* as well as alkane monooxygenase genes including *alkB, CYP153A*, and *almA* were amplified and sequenced as previously described (Sun et al., 2014b). Representative isolates of all phylotypes were selected for target gene detection. The PCR products were purified and directly sequenced using amplification primers (Sun et al., 2014b). The obtained sequences were searched against the NR database using BLAST (http://blast.ncbi.nlm.nih.gov/Blast.cgi), and the nearest reference sequences were retrieved. The derived amino acid sequences were aligned by CLUSTAL_X and manual correction. The phylogenetic tree was constructed after alignment using the neighbor-joining method (Saitou and Nei, 1987) in the MEGA 6.0 software (Tamura et al., 2013). Tree topology was evaluated with bootstrap analysis based on 1,000 resampling replicates.

### Detection of phenol- and alkane-degrading abilities

The isolates were first cultured in artificial sea water medium (ASW: 1.0 g yeast extract L^−1^, 5.0 g tryptone l^−1^, 4.0 g Na_2_SO_4_ l^−1^, 0.68 g KCl l^−1^, 0.1 g KBr L^−1^, 0.025 g H_3_BO_3_ l^−1^, 5.4 g MgCl_2_·H_2_O l^−1^, 1.5 g CaCl_2_·H_2_O l^−1^, 0.024 g SrCl_2_·6H_2_O l^−1^, 0.2 g NaHCO_3_ l^−1^, 0.2 g Na_2_HPO_4_ l^−1^, 0.5 g NH_4_Cl l^−1^, 0.002 g NaF l^−1^, 24 g NaCl l^−1^, 15 g agar l^−1^; pH 7.0-8.0) at 25°C with shaking at 150 rpm for 5 days. Cells were then harvested by centrifugation (2,000 *g* at 4°C for 10 min). Cell pellets were washed twice using sterile saline, and were suspended in an equal volume of saline. Then, 30 μl cells were inoculated into 3 ml MSM supplemented with 100 mg phenol l^−1^ (final concentration) or 100 mg hexadecane l^−1^ as sole carbon sources. Cell cultures were incubated at 30°C with shaking at 150 rpm in the dark, and cell growth was measured. After 5 days, cell cultures were sampled, and the concentrations of residual phenol and hexadecane were detected, as previously described (Sun et al., 2011).

### Sequence accession numbers

The 16S rRNA gene sequences of the clone library from contaminated soils were deposited into GenBank under the accession numbers KC763859-KC763888. The 16S rRNA gene sequences of the isolated strains were under the accession numbers KP711432-KP711625; the phenol hydroxylase genes and the flavin-binding monooxygenase gene *almA* were under the numbers KP711626-KP711653; the *alkB* and *CYP153A* genes were deposited into GenBank under the numbers KT160363-KT160415.

## Results

### Isolation of aerobic bacterial strains from soils under ambient and constant temperatures

In this work, a total of 595 bacterial isolates were isolated from the soils, including 335 isolates via S0 soil enrichments and 260 isolates via direct isolation from both soil S0 and S1. Among these, 336 and 259 bacterial species were obtained under ambient and constant temperatures, respectively.

**Figure 1:**
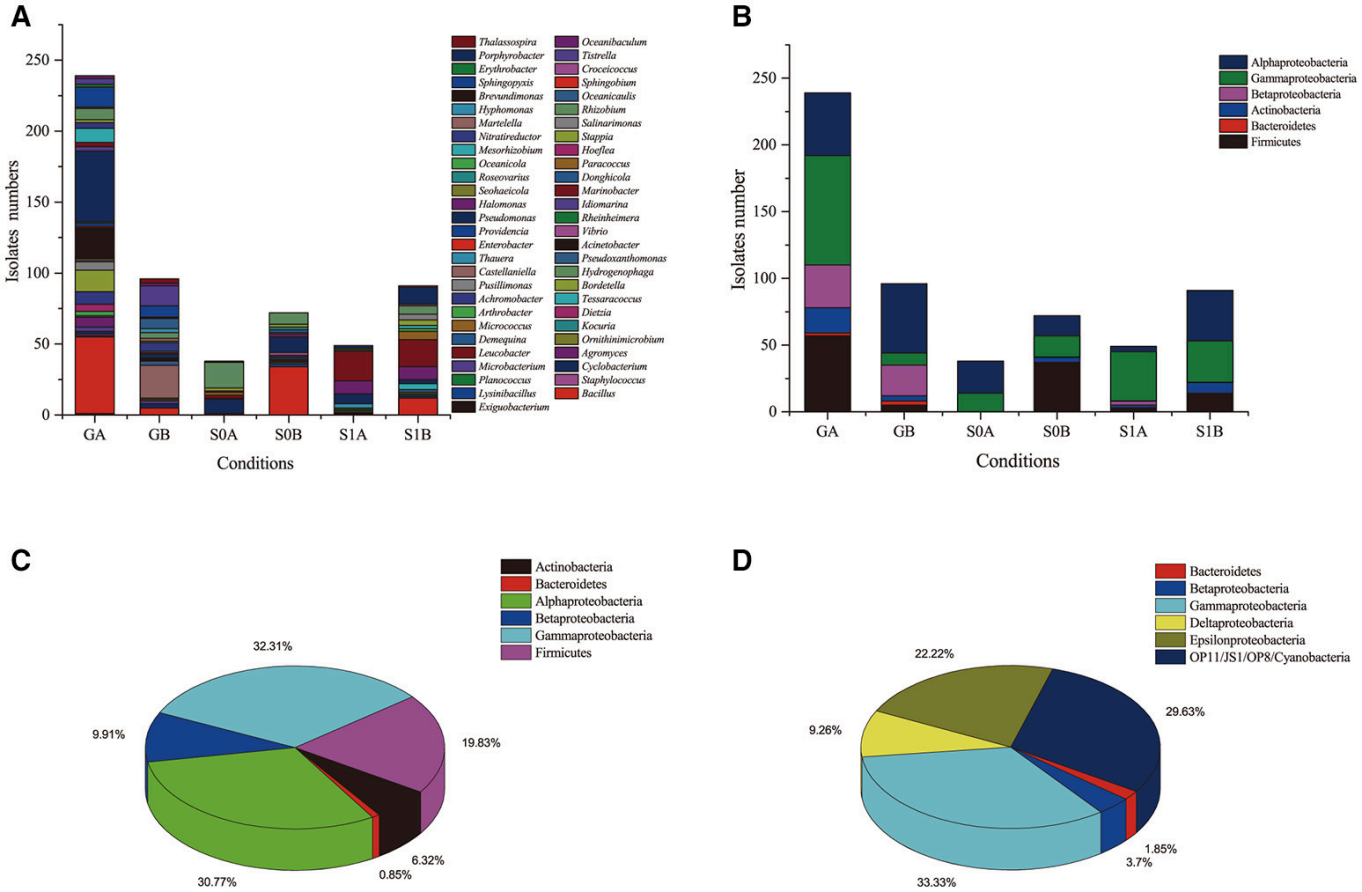
Composition fractions of isolates cultured in different conditions and from different soil samples on a **(A)** genus and **(B)** phylum levels. Bacterial composition obtained from of **(C)** cultured strains and **(D)** via the clone library method.

The isolates were distributed into four phyla (Actinobacteria, Firmicutes, Bacterioidetes, and Proteobacteria), and belonged to 56 genera; they were classified into 149 phylotypes. Proteobacteria was the most dominant phylum in the isolates. At the genus level, *Pseudomonas, Marinobacter*, and *Bacillus* were the dominant genera (Figure 1, Figure S1, Table **2**). According to colony morphology and phylogenetic analysis, 335 isolates obtained from “S0” soil using the enrichment method were affiliated into 39 genera and were classified into 109 phylotypes. It was notable that enrichment and isolation at ambient temperatures enhanced bacterial culturability. (i) The number of isolates obtained when incubated at ambient temperatures was much higher as compared with that when incubated at a constant temperature of 30°C. Ambient temperature enrichment yielded 239 isolates that were distributed into 31 genera; 96 isolates distributed into 22 genera were obtained from enrichment under constant temperature. (ii) Isolates cultured in ambient temperatures exhibited higher α-diversity as compared with those cultured at 30°C. The Shannon-Wiener index values for bacteria cultured at ambient temperature were all higher than 2.0, while those cultured at a constant temperature were < 2.0 (Table **2**); (iii) Higher number of unique genera (only obtained by one cultivating method) were obtained by incubating at ambient temperatures. We collected 17 unique genera, including *Achromobacter, Agromyces, Bordetella, Brachybacterium, Dietzia, Enterobacter, Erythrobacter, Hydrogenophaga, Hyphomonas, Idiomarina, Kocuria, Mycobacterium, Providencia, Pusillimonas, Rheinheimera, Staphylococcus*, and *Vibrio*, by incubating at ambient temperatures. Only seven unique genera were obtained by incubating at a constant temperature of 30°C. In addition, 15 genera were isolated under both culture conditions (Figure 1 and Figure S2). (iv) More novel or potential novel strains with lower 16S rRNA gene identities (< 98.6%) were obtained at ambient temperatures as compared with those obtained at 30°C (Table 1); two strains obtained at ambient temperatures have been published as type strains for novel species: *Negadavirga shengliensis* SLG210A2-5 (Hu et al., 2015) and *Glycocaulis albus* SLG210-30A1^T^ (Lv et al., 2014). These results suggested that ambient temperature cultures improve the culturability of bacteria from environmental samples.

**Table 1:** Basic information of strains isolated from different enrichment conditions.

	**S0**										**S1**			
	**G2**		**G3**		**G5**		**PW**		**MSM**		**PW**		**MSM**	
	**A**	**B**	**A**	**B**	**A**	**B**	**A**	**B**	**A**	**B**	**A**	**B**	**A**	**B**
Isolates	103	17	88	42	48	37	7	38	31	34	37	9	22	82
Phylotypes	35	7	20	10	22	15	6	22	11	12	21	7	8	38
Genus	17	6	13	8	15	13	4	12	8	8	10	5	5	19
n-Hexadecane degrader	43	1	29	7	16	24	5	22	16	13	22	3	15	28
n-Hexadecane degrader (%)	41.7	5.9	33.0	16.7	33.3	64.9	71.4	57.9	51.6	38.2	59.5	33.3	68.2	34.1
Novel or potential strains	7	1	2	2	6	1	0	1	0	0	1	0	0	7
Simpson index	0.79	0.79	0.87	0.67	0.85	0.84	0.71	0.76	0.67	0.70	0.76	0.81	0.80	0.91
Shannon-Wiener index	2.08	1.56	2.18	1.43	2.21	1.95	1.15	1.82	1.44	1.50	1.75	0.73	1.45	2.58

Furthermore, the effects of ambient/constant temperature differ between various taxa. For example, more number of Gammaproteobacteria and Betaproteobacteria were obtained by cultivating under the ambient temperatures: 8 *Betaproteobacteria* phylotypes (35 isolates, 6 genera) were isolated at ambient temperature, while only one phylotype (including 23 isolates) were obtained at 30°C. Similarly, a higher number of Gammaproteobacteria isolates were obtained at ambient temperature as compared with those obtained at a constant temperature. However, at the order level, *Pseudoxanthomonas* belonging to Xanthomonadales was only obtained from cultures incubated at 30°C (Figure S1).

A total of 110 and 150 isolates were obtained from S0 and S1 soils, respectively, using the direct-plating (without enrichment) method. These 260 isolates (27 genera) were affiliated with *Actinobacteria, Firmicutes*, and *Proteobacteria*, and were classified into 32 genera, and 45 phylotypes. In contrast to the enrichment method, more strains were isolated at constant temperature (163 isolates, 20 genera), rather than at ambient temperatures (97 isolates, 15 genera) using this method. (i) The α-diversities (Simpson index) of isolates cultured under ambient temperatures were lower as compared with those cultured under 30°C constant temperature (Table 1). (ii) More number of novel strains were obtained by culturing at a constant temperature (8 novel species) than were obtained by culturing at ambient temperatures (1 novel species) (Table 1). The strains directly isolated from the soil have been identified as novel taxonomic units, and include *Salinarimonas ramus* SL014B-41A4^T^ (Cai et al., 2011b), *Tessaracoccus oleiagri* SL014B-20A1^T^ (Cai et al., 2011b), *Halomonas shengliensis* SL014B-85^T^ (Wang et al., 2007a), *Halomonas gudaonensis* SL014B-69^T^ (Wang et al., 2007b), *Marinobacter gudaonensis* SL014B-61A^T^ (Gu et al., 2007), *Rubrimonas shengliensis* SL014B-28A2^T^ (Cai et al., 2011a), *Polymorphum gilvum* SL003B-26A1^T^ (Cai et al., 2011a) and *Nitratireductor shengliensis* SL014B-25A^T^ (Pan et al., 2014), and *Marinobacter shengliensis* SL013A-34A2^T^ (Luo et al., 2015).

### Isolation conditions affect the proportion of petroleum degraders

Hexadecane was able to be used as the sole carbon and energy source for growth of 244 isolates (Tables 1, 2). Using the enrichment method, 88 (36.8% of isolates) and 32 (33.0% of isolates) hexadecane-degrading isolates were obtained by incubating at ambient temperatures and a constant temperature, respectively. Specifically, using the G2 medium for enriching strains, 43 and one hexadecane-degrading isolates were obtained by the ambient and constant temperature method; the ratio of the number of hexadecane-degrading isolates obtained under ambient temperatures to that obtained under a constant temperature method were 29:7 and 16:24 for G3 and G5 media, respectively. Using the direct-plating method, 21 and 35 hexadecane-degrading isolates were obtained from S0 soil at ambient and constant temperatures, respectively; 37 and 31 hexadecane-degrading isolates were isolated from S1 soil at ambient and constant temperatures, respectively. It is worth noting that the number of hexadecane-degrading isolates obtained by culturing at ambient temperature were slightly higher than those obtained by culturing at a constant temperature. In addition, the direct plating method led to a higher ratio of hexadecane degraders as compared with that obtained via the enrichment method using G2 and G3 as enrichment media. Specifically, some isolates, such as *Pseudomonas* spp. SLG510A3-14 and SL014A-33A1-2, *Enterobacter* sp. SLG510A3-6, *Acinetobacter* spp. SLG310A2-9 and SL003A-18A1, *Nitratireductor* sp. SLG510B6-18, *Bacillus* sp. SLG210A2-18-1, *Rhizobium* sp. SL014B-37A2, *Labrenzia* sp. SL014B-4A2, *Paracoccus* sp. SL013B-8A1, *Planococcus* sp. SL013A-41A-2, *Halomonas* sp. SL014B-63A1-2, and *Marinobacter* sp. SL013A-25A demonstrated robust growth in MSM supplemented with hexadecane as the sole carbon source. Among them, nine strains were obtained from ambient-temperature cultures, and only six were obtained from constant-temperature cultures; five strains were obtained via the enrichment method, while eight strains were obtained via the direct-plating method. Furthermore, alkane-degrading ability was first detected in the *Enterobacter* and *Nitratireductor* strains.

**Table 2:** Degrading characteristics of isolated bacterial strains and the gene amplification of phenol-degrading gene and alkane-degrading genes.

**Strain**	**Closest type strains**	**Amount**	**GenBank No**.	**Identities (%)**	**Biodegradation**		**Genes**			
					**Phe**	**n-Hex^a^**	***pheN***	***alkB***	**P450**	***almA***
SLG510A3-4A2	*Pseudomonas songnenensis*	1	NR_148295	99.9	**–**	+	**–**	**–**	**–**	**–**
SLG210A2-34-3	*Pseudomonas songnenensis*	1	NR_148295	99.9	**–**	+	**–**	**–**	**–**	**–**
SLG310A2-2	*Pseudomonas songnenensis*	2	NR_148295	99.9	**–**	+	**–**	**–**	**–**	**–**
SLG310A2-40	*Pseudomonas songnenensis*	13	LT629797	99.0	**–**	**–**	**–**	+	**–**	**–**
SLG510A3-14	*Pseudomonas aeruginosa*	2	KT825518	100	**–**	+++++	**–**	**–**	**–**	**–**
SLG310A2-6A1-2	*Pseudomonas balearica*	3	NR_025972	99.7	+	+	**–**	**–**	**–**	**–**
SLG210B1-9A1	*Marinobacter alkaliphilus*	1	NR_112223	100	**–**	**–**	**–**	+	+	**–**
SLG510A10-4	*Marinobacter shengliensis*	3	KP711478.1	100	**–**	**–**	+	+	+	+
SLG210A2-10B2-2	*Providencia burhodogranariea*	2	NR_104914	99.4	**–**	++	**–**	**–**	**–**	**–**
SLG510A3-6	*Enterobacter asburiae*	1	CP011863	99.6	**–**	+++++	**–**	+	**–**	**–**
SLG310A2-9	*Acinetobacter venetianus*	16	NR_042049	100	**–**	++++	**–**	+	+	**–**
SLG510A10-22B1	*Acinetobacter venetianus*	1	NR_042049	100	**–**	++++	**–**	+	+	**–**
SLG510A3-11A1	*Acinetobacter venetianus*	2	NR_042049	100	**–**	++++	**–**	+	+	**–**
SLG510A6-3	*Acinetobacter venetianus*	2	NR_042049	100	**–**	++++	**–**	+	+	**–**
SLG510B10-21	*Acinetobacter venetianus*	2	NR_042049	100	**–**	++++	**–**	+	+	**–**
SLG310B1-12	*Pseudoxanthomonas mexicana*	2	NR_113973	100	**–**	+	**–**	**–**	**–**	**–**
SLG310B2-40A1	*Pseudoxanthomonas mexicana*	1	NR_113973	100	**–**	+	**–**	**–**	**–**	**–**
SLG210A2-35-2	*Hydrogenophaga palleronii*	1	NR_114132	99.1	**–**	+	**–**	**–**	+	**–**
SLG210A2-28A1	*Bordetella bronchiseptica*	2	NR_113628	98.1	**–**	+	**–**	**–**	**–**	**–**
SLG310A2-11A1	*Achromobacter pulmonis*	7	NR_117644	99.8	+	**–**	+	**–**	**–**	**–**
SLG510A3-26	*Achromobacter pulmonis*	2	NR_117644	99.8	+	**–**	+	**–**	**–**	**–**
SLG210A2-36-1	*Tistrella mobilis*	1	NR_114036	100	**–**	+	**–**	**–**	**–**	**–**
SLG210A3-2-1	*Tistrella mobilis*	1	NR_114036	100	**–**	+	**–**	**–**	**–**	**–**
SLG510A2-12-2	*Tistrella mobilis*	1	NR_114036	100	**–**	+	**–**	**–**	**–**	**–**
SLG510A10-23	*Tistrella mobilis*	1	NR_114036	100	**–**	+	**–**	**–**	**–**	**–**
SLG510B2-10	*Tistrella mobilis*	8	NR_114036	100	**–**	+	**–**	**–**	**–**	**–**
SLG510B10-1	*Tistrella mobilis*	5	NR_114036	100	**–**	+	**–**	**–**	**–**	**–**
SLG310A2-23	*Sphingopyxis chilensis*	1	NR_024631	99.6	**–**	**–**	**–**	**–**	+	**–**
SLG310B1-4-2	*Sphingopyxis chilensis*	4	NR_024631	99.6	**–**	**–**	**–**	**–**	+	**–**
SLG310B2-17	*Sphingopyxis chilensis*	4	NR_024631	99.6	**–**	**–**	**–**	**–**	+	**–**
SLG310A2-18B3	*Erythrobacter citreus*	2	NR_028741	99.4	**–**	**–**	**–**	**–**	+	**–**
SLG210A2-1-1	*Hyphomonas polymorpha*	3	KF863144	98.6	**–**	**–**	**–**	**–**	+	**–**
SLG310B2-7A2-2	*Brevundimonas diminuta*	1	NR_113602	99.9	+	**–**	+	**–**	**–**	**–**
SLG510B6-18	*Nitratireductor aquibiodomus*	1	NR_025262	99.6	**–**	+++++	**–**	+	**–**	**–**
SLG310A2-16	*Nitratireductor indicus*	4	NR_117518	100	**–**	+	**–**	**–**	**–**	**–**
SLG510B6-1	*Nitratireductor indicus*	2	NR_117518	100	**–**	+	**–**	**–**	**–**	**–**
SLG310A2-20	*Nitratireductor soli*	3	KP639570	99.9	**–**	+	**–**	**–**	**–**	**–**
SLG310B1-11	*Nitratireductor indicus*	1	NR_117518	97.8	**–**	+	**–**	**–**	**–**	**–**
SLG310B2-11	*Nitratireductor indicus*	1	NR_117518	97.8	**–**	+	**–**	**–**	**–**	**–**
SLG310B1-40A2	*Rhizobium halotolerans*	1	NR_125632	99.9	**–**	+	**–**	**–**	**–**	**–**
SLG310B2-4-1	*Rhizobium halotolerans*	1	NR_125632	99.9	**–**	+	**–**	**–**	**–**	**–**
SLG510A10-14B1	*Rhizobium halotolerans*	1	NR_125632	99.9	**–**	+	**–**	**–**	**–**	**–**
SLG510A3-33	*Rhizobium halotolerans*	1	NR_125632	99.9	**–**	+	**–**	**–**	**–**	**–**
SLG510A10-12	*Rhizobium helanshanense*	1	NR_133019	99.6	**–**	**–**	**–**	+	+	**–**
SLG210A2-38-1	*Agrobacterium fabrum*	1	NR_074266	100	**–**	++	**–**	**–**	**–**	**–**
SLG210B1-12	*Agrobacterium fabrum*	1	NR_074266	100	**–**	++	**–**	**–**	**–**	**–**
SLG510A10-7B2	*Agrobacterium fabrum*	1	NR_074266	100	**–**	++	**–**	**–**	**–**	**–**
SLG510B10-28	*Agrobacterium fabrum*	2	NR_074266	100	**–**	++	**–**	**–**	**–**	**–**
SLG210A6-6	*Exiguobacterium aestuarii*	1	NR_043005	99.5	**–**	+	**–**	**–**	**–**	**–**
SLG210A2-29-1	*Bacillus tianshenii*	11	NR_133704	99.8	**–**	+++	**–**	**–**	**–**	**–**
SLG210A3-15-4	*Bacillus tianshenii*	4	NR_133704	99.8	**–**	+++	**–**	**–**	**–**	**–**
SLG210A2-14C1	*Bacillus muralis*	5	NR_042083	99.9	**–**	+	**–**	**–**	**–**	**–**
SLG210A3-9-1	*Bacillus muralis*	1	NR_042083	99.9	**–**	+	**–**	**–**	**–**	**–**
SLG210A2-25-1	*Bacillus vietnamensis*	1	NR_113995	99.1	**–**	+	**–**	**–**	**–**	**–**
SLG210A2-2	*Bacillus idriensis*	1	NR_029022	99.7	**–**	+	**–**	**–**	**–**	**–**
SLG210A2-11	*Bacillus halosaccharovorans*	1	NR_109116	99.1	**–**	+	**–**	**–**	**–**	**–**
SLG210A2-18-1	*Bacillus litoralis*	3	NR_043015	98.5	**–**	++++	**–**	**–**	**–**	**–**
SLG210A3-8	*Bacillus litoralis*	3	NR_043015	98.5	**–**	++++	**–**	**–**	**–**	**–**
SLG510B10-40B1-2	*Microbacterium thalassium*	2	NR_042481	99.4	**–**	+	**–**	+	**-**	**-**
SLG310A2-4-1	*Agromyces italicus*	1	NR_043026	99.5	**–**	+	**–**	**–**	**–**	**–**
SLG510A2-27-2	*Kocuria turfanensis*	2	DQ531634	100	**–**	+	**–**	**–**	**–**	**–**
SLG510B2-13A2	*Arthrobacter tumbae*	1	NR_042078	99.7	**–**	+	**–**	**–**	+	**–**
SLG210A6-7	*Arthrobacter protophormiae*	2	NR_026195	100	**–**	+	**–**	**–**	**–**	**–**
SLG210A2-38-2	*Arthrobacter protophormiae*	1	NR_026195	100	**–**	+	**–**	**–**	**–**	**–**
SLG510B10-2-2	*Micrococcus terreus*	1	NR_116649	100	**–**	+	**–**	**–**	**–**	**–**
SL003A-1A2	*Rhizobium rosettiformans*	1	NR_116445	100	**–**	+	**–**	**–**	**–**	**–**
SL003B-10A2	*Rhizobium selenitireducens*	6	NR_044216	99.0	**–**	+	**–**	**–**	**–**	**–**
SL004A-8A1	*Rhizobium rosettiformans*	14	NR_116445	100	**–**	+	**–**	**–**	**–**	**–**
SL014A-30A	*Rhizobium rosettiformans*	1	NR_116445	100	**–**	+	**–**	**–**	**–**	**–**
SL014B-16A1-1	*Rhizobium selenitireducens*	1	NR_044216	99.0	**–**	+	**–**	**–**	**–**	**–**
SL014B-37A2	*Rhizobium tarimense*	3	NR_117850	98.8	**–**	++++	**–**	**–**	**–**	**–**
SL014B-87A3	*Stappia indica*	1	NR_116431	99.8	**–**	++	**–**	**–**	**–**	**–**
SL003B-19A3	*Polymorphum gilvum*	4	NR_074240	100	**–**	+	**–**	**–**	**–**	**–**
SL014B-4A2	*Labrenzia aggregata*	1	NR_113861	99.7	**–**	+++	**–**	**–**	**–**	**–**
SL014B-40A2	*Stappia meyerae*	1	NR_115949	98.8	+	+	**–**	**–**	**–**	**–**
SL004A-4A	*Stappia meyerae*	1	NR_115949	98.8	+	+	**-**	**-**	**-**	**-**
SL014B-56A3-1	*Paracoccus homiensis*	4	NR_043733	99.5	**–**	+	**–**	**–**	**–**	**–**
SL013B-8A1	*Paracoccus marcusii*	1	NR_044922	100	**–**	+++	+	**–**	**–**	**–**
SL014B-90A-2	*Salinarimonas rosea*	3	NR_116487	98.6	**–**	++	**–**	**–**	**–**	**–**
SL014B-17A3	*Porphyrobacter donghaensis*	1	NR_025816	99.8	**–**	**–**	+	**–**	**–**	**–**
SL013B-2A1-3	*Porphyrobacter donghaensis*	1	NR_025816	99.8	**–**	**–**	+	**–**	**–**	**–**
SL013A-50A	*Porphyrobacter colymbi*	2	NR_114328	99.4	**–**	+	**–**	**–**	**–**	**–**
SL014A-10A1	*Porphyrobacter colymbi*	9	NR_114328	99.4	**–**	+	**–**	**–**	**–**	**–**
SL013A-40A1	*Arthrobacter tumbae*	1	NR_042078	99.9	**–**	+	**–**	**–**	**–**	**–**
SL013A-41A-2	*Planococcus antarcticus*	3	CP016534	99.0	**–**	+++++	**–**	**–**	**–**	**–**
SL003B-7A-2	*Planococcus antarctic*us	1	CP016534	99.0	**–**	+++++				
SL014B-56A1-1	*Planococcus antarcticus*	1	CP016534	99.0	**–**	+++++	**–**	**–**	**–**	**–**
SL014B-4A1	*Lysinibacillus alkalisoli*	1	KX258757	99.6	**–**	+	**–**	**–**	**–**	**–**
SL003B-20A2	*Lysinibacillus fusiformis*	1	NR_112569	99.9	**–**	+	**–**	**–**	**–**	**–**
SL004B-24A	*Lysinibacillus fusiformis*	1	NR_112569	99.9	**–**	+	**–**	**–**	**–**	**–**
SL004B-4A	*Bacillus megaterium*	5	KJ476721	99.7	**–**	+	**–**	**–**	**–**	**–**
SL014B-7A1-2	*Bacillus megaterium*	1	KJ476721	99.7	**–**	+	**–**	**–**	**–**	**–**
SL003B-7B1-1	*Bacillus subterraneus*	2	NR_104749	99.4	**–**	+	**–**	**–**	**–**	**–**
SL013B-14A3	*Bacillus stratosphericus*	1	NR_118441	100	**–**	++	**–**	**–**	**–**	**–**
SL003B-30B1	*Bacillus stratosphericus*	1	NR_118441	100	**–**	++	**–**	**–**	**–**	**–**
SL014B-20A2	*Bacillus stratosphericus*	1	NR_118441	100	**–**	++	**–**	**–**	**–**	**–**
SL003B-19A2	*Bacillus mojavensis*	5	NR_118290	100	**–**	+	**–**	**–**	**–**	**–**
SL003B-11B2-2	*Bacillus aquimaris*	1	NR_025241	99.5	**–**	**-**	**–**	**–**	**–**	**–**
SL013A-42A1	*Thauera selenatis*	1	NR_026474	98.5	+	**–**	**–**	**–**	**–**	**–**
SL013A-36A-1	*Thauera chlorobenzoica*	2	CP018839	99.0	+	+	**–**	**–**	**–**	**–**
SL004B-31A1	*Vibrio fluvialis*	2	CP014035	99.9	**–**	+	**–**	**–**	**–**	**–**
SL014B-21B1-2	*Pseudomonas stutzeri*	1	CP002881	100	+	++	+	**–**	**–**	**–**
SL004A-44A	*Pseudomonas stutzeri*	6	CP002881	100	**–**	**–**	+	**–**	**–**	**–**
SL004B-21B1-1	*Pseudomonas stutzeri*	6	CP002881	100	**–**	**–**	+	**–**	**–**	**–**
SL003B-18B2-1	*Pseudomonas stutzeri*	2	CP002881	100	**–**	**–**	+	**–**	**–**	**–**
SL003A-2A1-2	*Pseudomonas xanthomarina*	2	NR_041044	99.1	**–**	+	**–**	**–**	**–**	**–**
SL003B-7A-3	*Pseudomonas japonica*	1	KT825519	99.9	**–**	+	+	**–**	**–**	**–**
SL004B-28A-3	*Pseudomonas japonica*	1	KT825519	99.9	**–**	+	+	**–**	+	**–**
SL014B-42A3	*Pseudomonas japonica*	2	KT825519	99.9	+	++	+	**–**	**–**	**–**
SL003A-18A1	*Acinetobacter indicus*	1	NR_117784	100	**–**	++++	**–**	**–**	+	**–**
SL014B-63A1-2	*Halomonas ventosae*	1	NR_042812	99.7	**–**	+++++	**–**	**–**	**–**	**–**
SL014A-11A2	*Halomonas ventosae*	3	NR_042812	99.9	**–**	++	**–**	**–**	**–**	**–**
SL013A-32A1	*Halomonas ventosae*	1	NR_042812	99.9	**–**	+++	**–**	**–**	**–**	**–**
SL004A**-**30A	*Halomonas ventosae*	1	NR_042812	99.9	**–**	+++	**–**	**–**	**–**	**–**
SL014A-23A	*Halomonas campaniensis*	1	NR_042157	99.6	**–**	++	**–**	**–**	**–**	**–**
SL013A-18A	*Halomonas meridiana*	1	NR_042066	99.3	**–**	**–**	+	**–**	**–**	**–**
SL013A-7A1	*Halomonas zhaodongensis*	2	NR_125612	99.7	**–**	++	**–**	**–**	**–**	**–**
SL004B-23A3-1	*Halomonas zhaodongensis*	1	NR_125612	99.7	**–**	++	**–**	**–**	**–**	**–**
SL013B-12A1-3	*Halomonas zhaodongensis*	1	NR_125612	99.7	**–**	++	**–**	**–**	**–**	**–**
SL014B-11A2*	*Marinobacter gudaonensis*	9	DQ414419	100	**–**	**–**	**–**	+	+	+
SL014A-2A1	*Marinobacter flavimaris*	2	NR_025799	99.5	**–**	**–**	**–**	+	+	+
SL013A-35A	*Marinobacter flavimaris*	2	NR_025799	99.5	**–**	**–**	**–**	+	+	+
SL014B-80A2	*Marinobacter hydrocarbonoclasticus*	2	NR_074619	100	**–**	**–**	+	+	+	+
SL014A-10A1-2	*Marinobacter hydrocarbonoclasticus*	2	NR_074619	100	**–**	**–**	+	+	+	**–**
SL013A-7A2	*Marinobacter hydrocarbonoclasticus*	2	NR_074619	100	**–**	**–**	+	+	+	**–**
SL004A-39A	*Marinobacter hydrocarbonoclasticus*	1	NR_074619	99.9	**–**	**–**	**–**	+	**–**	+
SL014B-45A-2	*Marinobacter shengliensis*	1	KP711478	100	+	**–**	+	+	+	+
SL013B-15A5	*Marinobacter shengliensis*	3	KP711478	100	+	**–**	+	+	+	**–**
SL013A-34A-2*	*Marinobacter shengliensis*	1	KP711478	100	**–**	++	**–**	+	+	+
SL003A-19A-1	*Marinobacter shengliensis*	1	KP711478	100	**–**	+	**–**	+	+	**–**
SL013A-38A	*Marinobacter alkaliphilus*	1	NR_112223	99.7	**–**	**–**	**–**	+	**–**	+
SL013A-24A	*Marinobacter shengliensis*	1	KP711478	100	+	**–**	**–**	+	+	+
SL013A-25A	*Marinobacter shengliensis*	10	KP711478	100	**–**	++++	**–**	+	+	+
SL004B-10A-2	*Donghicola xiamenensis*	2	NR_043565	98.1	**–**	+				
SL014B-22A1	*Labrenzia alba*	1	NR_042378	99.0	**–**	+	**–**	**–**	**–**	**–**
SL014B-55A2	*Rhizobium tarimense*	2	NR_117850	100	**–**	+	**–**	**–**	**–**	**–**
SL004B-4A	*Bacillus megaterium*	1	KJ476721	99.7	**–**	+	**–**	**–**	**–**	**–**
SL014B-14A2	*Microbacterium saccharophilum*	1	NR_114342	99.5	**–**	+	**–**	**–**	**–**	**–**
SL003B-19B2-1	*Leucobacter komagatae*	1	NR_114929	100	**–**	+	**–**	**–**	**–**	**–**
SL003B-1A8	*Pseudoxanthomonas mexicana*	1	NR_113973	100	**–**	+	**–**	**–**	**–**	**–**
SL004B-16A2-3	*Pseudomonas stutzeri*	1	CP002881	99.7	+	**–**	+	**–**	**–**	**–**
SL013B-15A2	*Marinobacter shengliensis*	1	KP711478	99.9	**–**	**–**	**–**	**–**	+	+
SL014B-81A3-1	*Marinobacter segnicrescens*	1	NR_044124	100	**–**	+	**–**	**–**	**–**	**–**
SL014B-23A1-1	*Marinobacter flavimaris*	3	NR_025799	99.4	**–**	**–**	**–**	+	**–**	+
SL014B-22A2-2	*Ornithinimicrobium pekingense*	1	NR_043886	99.7	**–**	+++	**–**	**–**	**–**	**–**
SL014A-33A1-2	*Pseudomonas graminis*	1	NR_026395	98.9	**–**	+++++	**–**	+	+	**–**

a growth ability of the strains with n-hexadecane in MSM medium, +++++, ++++, +++, ++, + and –, indicating the growth capability from strong to weak with n-hexadecane as sole carbon and energy source, measured by optical density at 600 nm. +++++, growth (OD_600_ > 0.5) after a 7-day incubation at 30°C; ++++, growth (0.5 > OD_600_ > 0.4) after a 7-day incubation at 30°C; +++, growth (0.4 > OD_600_ > 0.3); ++, growth (0.3 > OD_600_ > 0.2) after a 7-day incubation at 30°C; +, growth (0.1 < OD_600_ < 0.2) after a 7-day incubation at 25°C; –, no growth.

* *strain is the type strain*.

Results indicated that 27 isolates were capable of degrading phenols. Among them, 18 isolates were obtained from ambient temperature cultures, and 9 isolates were obtained from constant temperature cultures. All phenol-degrading bacterial strains were assigned to Proteobacteria: one *Brevundimonas* and two *Stappia* isolates belonging to Alphaproteobacteria, three *Thauera* and nine *Achromobacter* isolates belonging to Betaproteobacteria, as well as five *Marinobacter* and seven *Pseudomonas* isolates belonging to Gammaproteobacteria (Table 2). These were some differences in the taxonomy of phenol-degrading strains isolated from oil-production water; all phenol-degrading strains belonged to *Betaproteobacteria* (Sun et al., 2014b).

### Distribution of *pheN, alkB*, CYP153A, and *almA* genes in isolates

**Figure 2:**
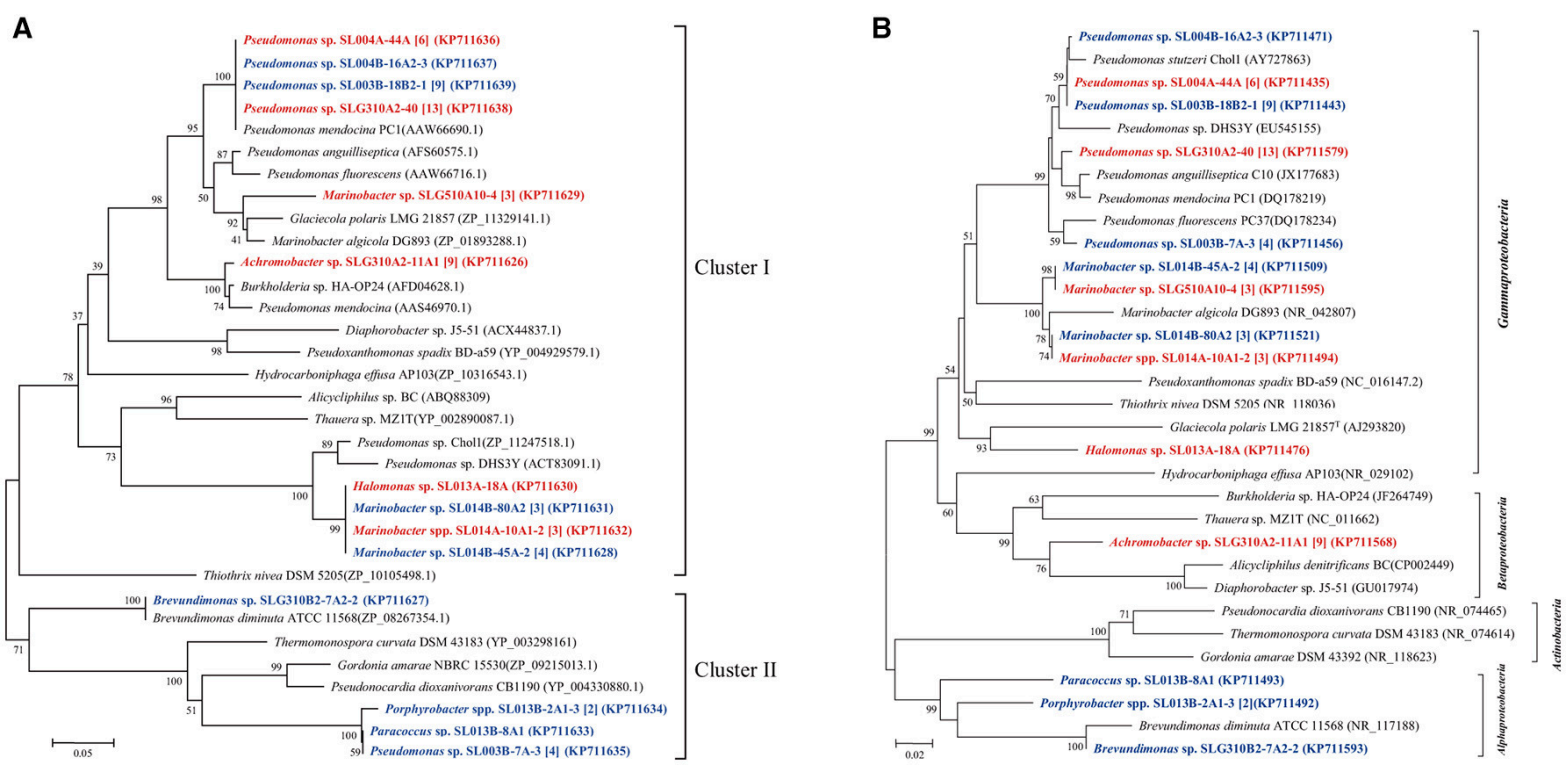
Phylogenetic trees were constructed based on partial amino sequences of **(A)** phenol hydroxylase and **(B)** 16S rRNA gene sequence of bacterial strains obtained from the oil-contaminated soils. Tree topology was evaluated by bootstrap analysis based on 1,000 resampling replicates. Bootstrap values (%) are indicated at the nodes; only values > 50 are shown. The strains isolated at ambient temperature were indicated by bolded red letters; isolates cultured at 30°C were indicated by bold letters in blue; numbers in square brackets are the number of isolates within this phylotype. Scale bars represent 0.05 substitutions per site.

Phenol hydroxylase, which catalyzes the initial step in most phenol catabolic pathway, is usually used to evaluate the phenol-degrading ability of bacterial strains (Qian et al., 1997; Sun et al., 2012, 2014a). The *pheN* gene was detected in 60 isolates, including 35 isolates (6 phylotypes; 10.7% of total isolates) in ambient-temperature cultures and 25 isolates (8 phylotypes; 9.6% of total isolates from constant temperature) in constant-temperature cultures. Conversely, more number of strains harboring the *pheN* gene were isolated by the direct-plating method (34 isolates, 11 phylotypes; 13.6% of the total strains) as compared with that isolated by the enrichment method (26 isolates, 4 phylotypes; 7.7% of the total strains). These *pheN* genes were categorized into two clusters according to their phylogenetic distance (Figure 2). Cluster I contained 52 sequences (4 genotypes) from 52 isolates (10 phylotypes) belonging to *Marinobacter, Pseudomonas, Achromobacter*, and *Halomonas*. Among these, *Achromobacter* belonged to *Betaproteobacteria*, while the others belonged to *Gammaproteobacteria*. Gene sequences in Cluster I were closely related to *pheN* from the *Pseudomonas* strains. Cluster II contained 8 sequences (3 genotypes) from 4 phylotypes (8 isolates) belonging to *Pseudomonas, Paracoccus, Porphyrobacter*, and *Brevundimonas*; this cluster was closely related to those belonging to Alphaproteobacteria and Actinobacteria. We noted that different cultivating methods may result in different genes; strains harboring the Cluster II *pheN* genes in the present study were only obtained from constant temperature cultures, while all strains obtained from ambient temperature cultures and a few strains obtained from constant temperature cultures contained Cluster I *pheN* genes. Additionally, although the *pheN* gene was detected in *Marinobacter* sp. SLG510A10-4, *Paracoccus* sp. SL013B-8A1, *Porphyrobacter* sp. SL014B-17A3, and *Halomonas* sp. SL014B-63A1-2 and SL013A-18A, phenol-degrading abilities were not detected in these strains (Figure 2).

Terminal hydroxylation of alkanes yields the corresponding primary alcohols, and it is a crucial step that significantly enhances the hydrophilicity of substrates (Nie et al., 2011, 2014a,b). In bacteria, this reaction is catalyzed by different enzymes, including those encoded by *almA, CYP153A*, and *alkB/alkM*. A total of 27 unique *CYP153A* sequences were derived from 91 bacterial isolates that span over 13 genera; 24 unique *alkB*/*alkM* sequences were derived from 93 isolates in nine genera, and 14 *almA* sequences were obtained from 34 isolates in two genera.

**Figure 3:**
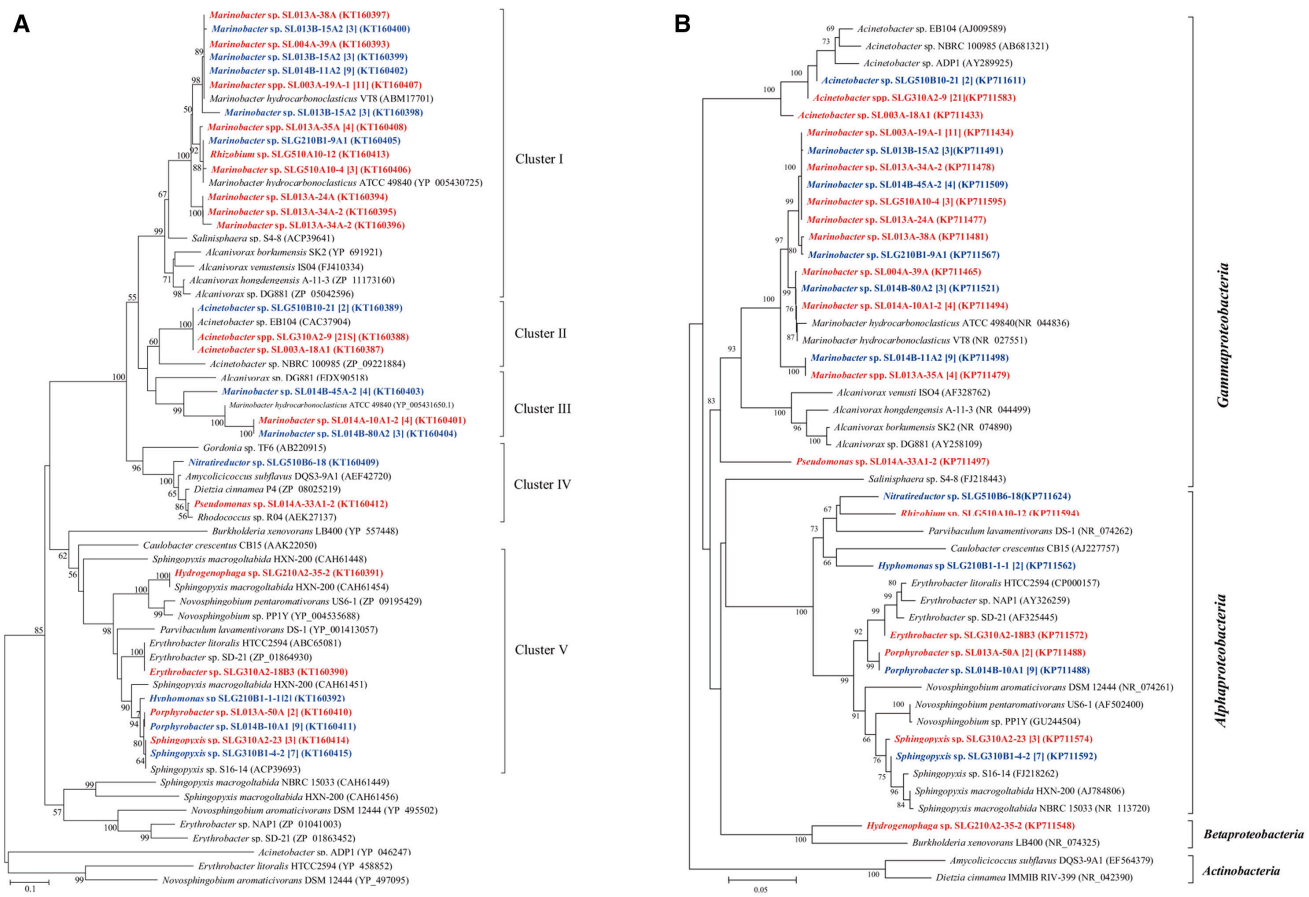
Phylogenetic trees were constructed based on the partial amino sequences of the **(A)** P450 (CYP153A) and **(B)** 16S rRNA gene sequences of bacterial strains which were obtained from the oil-contaminated soils. The strains isolated at ambient temperature were indicated by bolded red letters; isolates cultured at 30°C were indicated by bold letters in blue; numbers in square brackets are the number of isolates within this phylotype. The scale bar represents 0.1 substitutions per site.

The sequences of *CYP153A*, encoding a medium-chain-length alkane hydroxylase obtained from 91 isolates, were categorized into five clusters (Figure 3). As the biggest cluster, Cluster I contained 43 sequences (6 genotypes) from 36 isolates (11 phylotypes) belonging to *Marinobacter* and *Rhizobium*. Among them, three and two *CYP153A* sequences are present in *Marinobacter* spp. SL013B-15A2 and SL013A-34A-2, respectively. Cluster II included a unique sequence from 24 *Acinetobacter* isolates, which is closely related to *Acinetobacter* sp. NBRC100985 (ZP09221884). Cluster III consisted of two unique sequences from 11 *Marinobacter* isolates, which were closely related to *Alvanivorax* sp. DG881 (EDX90518) and *Marinobacter hydrocarbonoclasticus* ATCC 49840 (YP005431650) (Grimaud et al., 2012). Cluster IV was composed of P450 *CYP153A* genes from *Amycolicicoccus subflavus* DQS3-9A1^T^ (Nie et al., 2013). This cluster consisted of only two unique sequences from two distinct genera: *Nitratireductor* belonging to Alphaproteobacteria, and *Pseudomonas* belonging to Gammaproteobacteria. Cluster V consisted of seven unique sequences from 25 isolates, which belonged to *Hydrogenophaga (*Betaproteobacteria*), Erythrobacter, Hyphomonas, Sphingopyxis*, and *Porphyrobacter*; these genes clustered with the *CYP153* genes from strains belonging to Alphaproteobacteria. The ratio of the number of *CYP153A*-harboring isolates in ambient-temperature cultures to that in constant-temperature cultures was 24:13 (8:3 phylotypes), 22:2 (2:1), 0:7 (0:2), 1:1 (1:1), and 7:18 (4:3) in Cluster I, II, III, IV, and V, respectively, suggesting that ambient temperature may benefit isolation of stains containing the *CYP153A* gene. Furthermore, the method of isolation also somewhat affected the type of strains isolated. The ratio of the number of *CYP153A*-harboring isolates obtained by the enrichment method to that obtained by direct-plating method was 33:4 (8:4), 22:2 (2:1), 1:1 (1:1), 0:7 (0:2), and 14:11 (5:2) in cluster I, II, III, IV, and group II, respectively. Additionally, horizontal gene transfer was found in *CYP153A* genes. For example, *CYP153A* sequences from the *Rhizobium* sp. SLG510A10-12 and *Marinobacter* sp. SLG210B1-9A1 strains had identical *CYP153A* genes, and clustered together in the phylogenetic tree; this was inconsistent with the 16S rRNA gene tree (Figure 1). Furthermore, strains harboring Cluster I, Cluster IV, as well as Cluster V *CYP153A* genes did not grow in MSM supplemented with hexadecane as the sole carbon source. Interestingly, most of strains harboring the Cluster II and IV *CYP153A* gene were obtained by the enrichment method (Figure 3).

**Figure 4:**
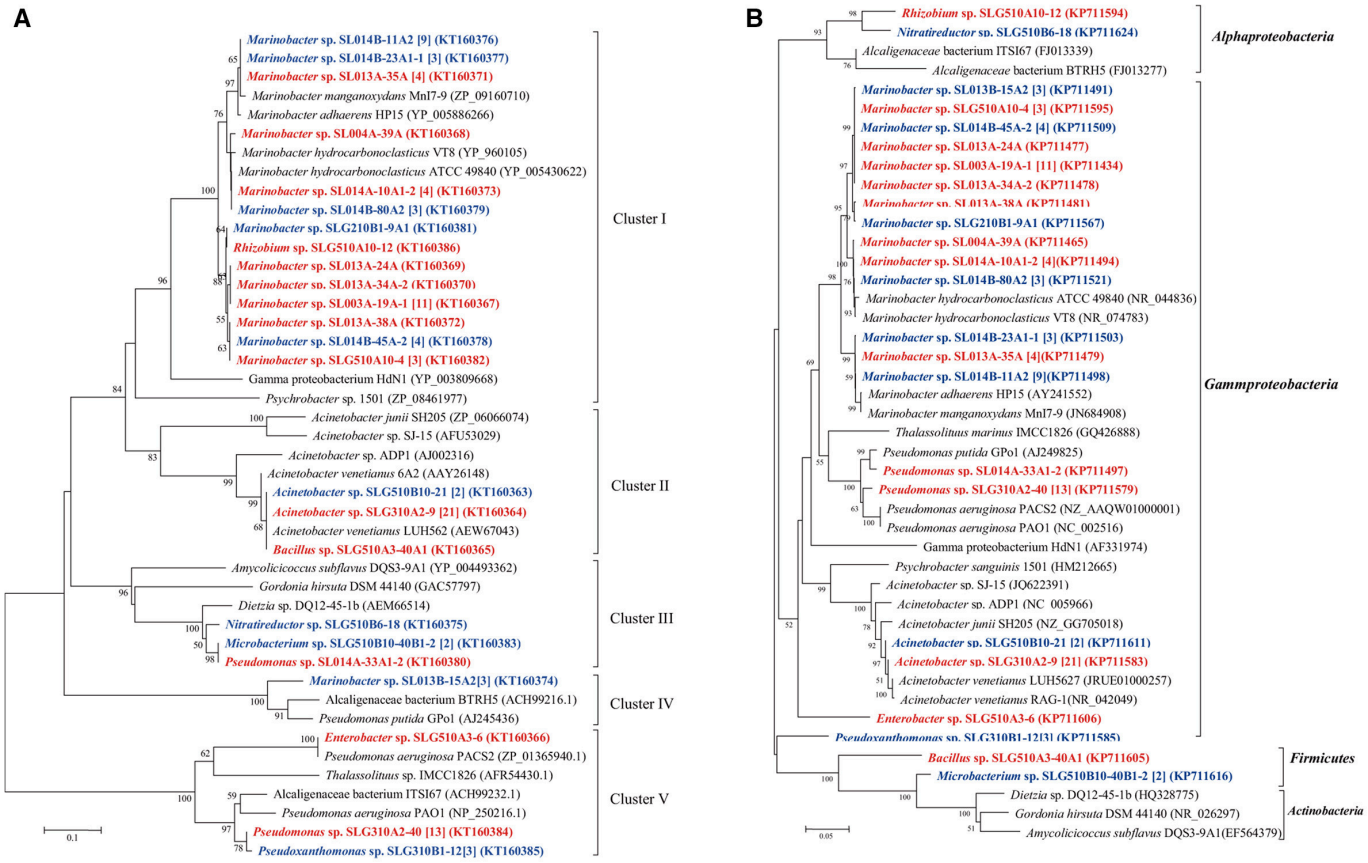
Phylogenetic tree was constructed based on the partial amino sequences of **(A)** alkane monooxygenase (AlkB) and **(B)** 16S rRNA gene sequences of bacterial strains obtained from oil-contaminated soils. The scale bar represents 0.1 substitutions per site. Acinetobacter venetianus 6A2 has the same 16S rRNA as Acinetobacter venetianus RAG-1 (Throne-Holst et al., 2006).

In total, 13 unique medium-length-chain hydroxylase *alkB/alkM* genes, an integral-membrane non-heme diiron monooxygenase, were obtained from 93 isolates and categorized into four clusters (Figure 4). As the biggest cluster, cluster I comprised of six unique sequences clustered with *alkB*/*alkM* genes from *Marinobacter hydrocarbonoclasticus* VT8 (Singer et al., 2011). Among these sequences, only one sequence was obtained from *Rhizobium* sp. SLG510A10-12, the others were obtained from *Marinobacter* strains. Genes in cluster II, which clustered with *Acinetobacter* sp. ADP1, consisted only of one unique sequence from three distinct genera: *Acinetobacter, Bacillus*, and *Microbacterium*, which belonged to Gammaproteobacteria, *Firmicutes*, and *Actinobacteria*, respectively. Cluster III contained two unique sequences from 24 isolates belonging to *Nitratireductor* (Alphaproteobacteria), *Microbacterium* (Actinobacteria), and *Pseudomonas* (Gammaproteobacteria) strains. This cluster was usually detected in strains belonging to Actinobacteria, in the *Dietzia* genus (Sun et al., 2014b). Cluster IV had only a single unique sequence from three *Marinobacter* isolates obtained at a constant temperature, and it showed the highest identity to the *alkB* gene in *Pseudomonas* sp. GPo1 (van Beilen et al., 2001). Cluster V consisted of three unique sequences from 17 isolates belonging to two *Gammaproteobacteria* genera: *Enterobacter* and *Pseudomonas*. Similar to the case of *CYP153A* gene, cultivation mode also somewhat affected the clusters of *alkB/alkM* genes. (i) The ratio of the number of *alkB/alkM*-harboring isolates by direct-plating method to that by enrichment methods was 42:5 (11:3), 0:24 (0:4), 1:3 (1:3), 3:0 (1:0), and 0:17 (0:3) in Cluster I, II, III, IV, and V, respectively. The direct-plating method resulted in more isolates (strains) harboring sequences in Cluster I and IV, while the enrichment method resulted in strains harboring *alkB/alkM* in Cluster II and IV. (ii) The ratio of the number of strains obtained under ambient temperatures to that obtained under a constant temperature was 27:20 (9:5), 22:5 (3:2), 1:3 (1:2), 0:3 (0:1), and 14:6 (2:2) in Cluster I, II, III, IV, and V, respectively, suggesting that ambient temperatures promote isolation of strains harboring cluster II and Cluster V *alkB/alkM* genes, while a constant temperature results in higher number of isolates containing *alkB/alkM* genes in Cluster III and IV. For the first time, an alkane monooxygenase gene and hexadecane-degrading ability were detected in *Nitratireductor* species. (Figure 4) More interestingly, among the strains harboring Cluster I and V *alkB*/*alkM* genes, only strains *Marinobacter* sp. SL013A-34A-2 harboring *alkB* gene of Cluster I and *Enterobacter* sp. SLG510A3-6 could utilize alkanes as the sole carbon source for growth; no obvious growth was detected in other strains. In contrast, four of the six strains harboring Cluster II genes, and all three strains harboring Cluster III *alkB*/*alkM* demonstrated significant growth under the same culture conditions. It was interesting to note that most of the strains harboring Cluster II and III *alkB*/*alkM* were obtained via the enrichment method (Figure 4).

**Figure 5:**
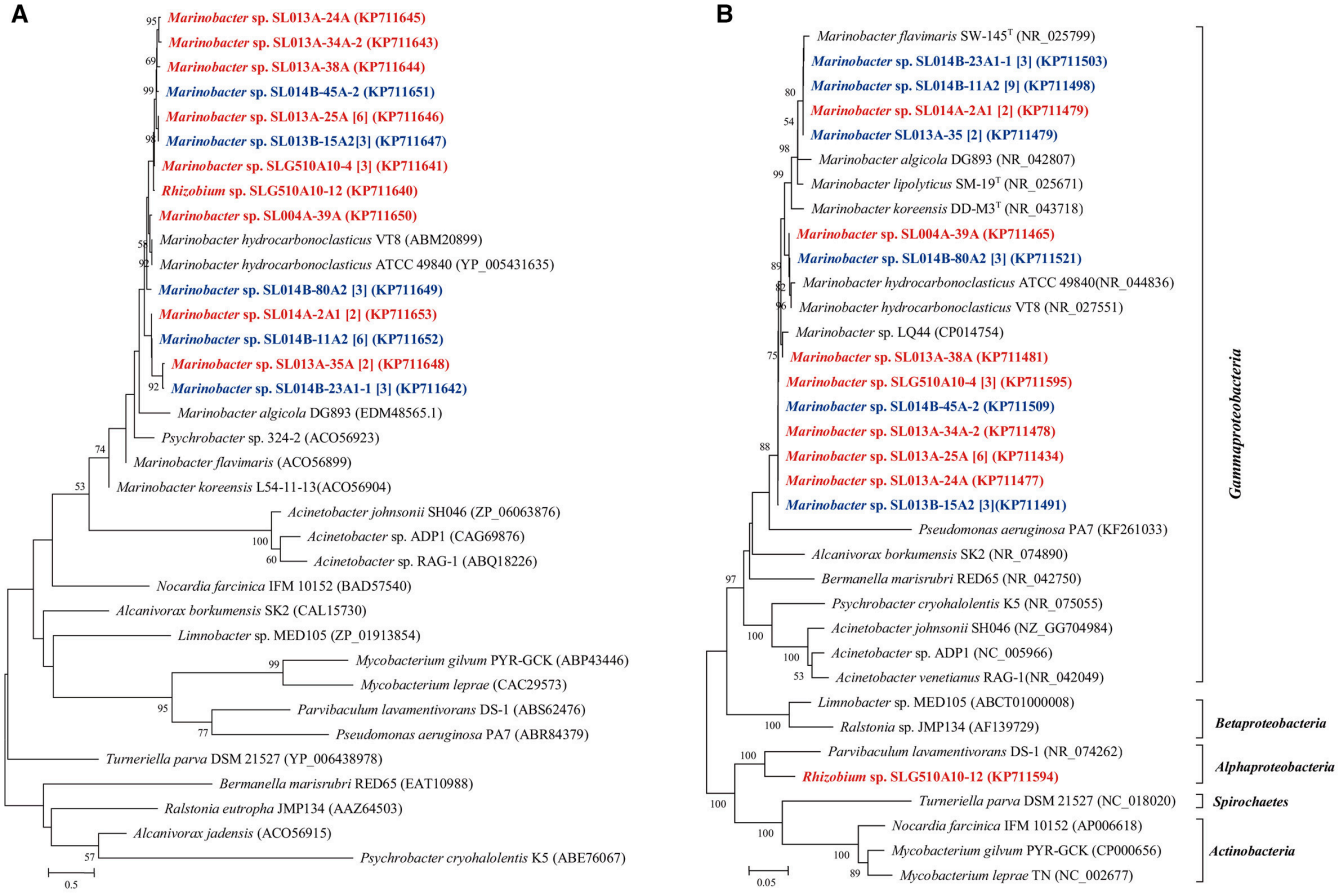
Phylogenetic trees were constructed based on the partial amino sequence of **(A)** alkane monooxygenase (AlmA) and **(B)** the 16S rRNA gene sequences of bacterial strains obtained from the oil-contaminated soils. The scale bar represents 0.5 and 0.05 substitutions per site.

Another putative monooxygenase of the flavin-binding family AlmA catalyzes the metabolism of long-chain *n*-alkanes. In the present study, a total of 12 unique *almA* sequences were detected in 36 isolates belonging to two genera: *Marinobacter* and *Rhizobium*. These genes were all closely related to those from *Marinobacter* strains regardless of their distinct taxonomic position. We found only 5.5% (19 isolates) of ambient-temperature isolates were contained the *almA* genes, while 6.1% of constant-temperature (17 isolates) isolates harbored the same genes (Figure 5).

It was observed that the *Marinobacter* strains can express multiple alkane hydroxylase genes. For example, three kinds of alkane monooxygenase genes including *alkB, CYP153A, alma*, and phenol hydroxylase genes were simultaneously detected in *Marinobacter* spp. SLG510A10-4, SL014B-80A2, and SL014B-45A-2. *Marinobacter gudaonensis* SL014B-11A2^T^, *Marinobacter* spp. SL013A-35A, SL004A-39A, SL013A-34A-2, SL013A-38A, SL013A-24A, SL013A-25A, and SL013B-15A2 all expressed *alkB, CYP153A*, and *almA* genes. Among them, strain SL013A-34A-2 had two copies of the *CYP153A* gene. In addition, *Marinobacter* sp. SLG210B1-9A1 harbored both *alkB* and *CYP153A* genes, and *CYP153A* and *almA* genes were simultaneously detected in *Marinobacter* sp. SL014B-23A1-1. Furthermore, three copies of the *CYP153A* gene were detected in *Marinobacter* sp. SL013-15A2. However, most of these strains did not grow in the MSM supplemented with hexadecane.

## Discussion

The clone library result showed that the bacterial community in oil-contaminated soils is comprised of species belonging to Proteobacteria (69.1%), Cyanobacteria (1.8%), Bacteroidetes (1.8%), and several unknown bacterial species (27.0%); no strains belonging to Actinobacteria and Firmicutes were detected. Proteobacteria in oil-contaminated soil consisted of Gammaproteobacteria, Epsilonproteobacteria, Alphaproteobacteria, Betaproteobacteria, and Deltaproteobacteria. Among them, the proportion of Gammaproteobacteria and Epsilonproteobacteria were much greater as compared with that of the three other subdivisions of Proteobacteria (Figure 1D). It is surprising that some popular oil-degrading species in the environment such as *Pseudomonas* and *Dietzia* strains (Tang et al., 2012; Gharibzahedi et al., 2014) were not detected with the clone library method. Isolation of bacterial strains from the bacterial community in the soil yielded very different result as compared with that from the cultivated method. For example, *Actinobacteria* and *Firmicutes* strains were isolated from the two soil samples, but were not detected in the colony library. It has been reported that the rare biosphere taxa in a given environmental have important ecological roles and are reservoirs of genetic and functional diversity (Lynch and Neufeld, 2015). They may become dominant under optimal conditions. Our study suggested the activation and rapid growth of rare species during culture. Previous studies also demonstrated that the culture-dependent and culture-independent methods provided two extremely divergent microbial communities: nearly 40% isolated strains from oil-contaminated soil were unable to be detected by high-throughput sequencing method (M'rassi et al., 2015). It was found that most of the shared operational taxonomic units (OTUs) between the two isolation methods belonged to the *Sphingobium* and *Sphingomonas* (Sphingomonadales), and *Pseudomonas* (Pseudomonadales) and *Arthrobacter* (Actinomycetales) genera.

Along with application of culture-independent methods in microbial ecology, it is recognized that most of the strains in the environment are uncultured, and are difficult to culture with the usual methods; these are known as uncultured strains (Zengler et al., 2002). It is believed that cultural media, temperature, pressure, antioxidant, and carbon and nitrogen sources could affect the isolation of microbes from various environments (Gartner et al., 2011; Sun et al., 2014b). Results from the present study also suggested that different isolating conditions, including carbon source, incubation temperature, and concentration of carbon affect the diversities of isolates. Among these factors, temperature is one the most important factors affecting the culturability of bacteria from the environments. According to their optimal temperature, bacteria could be subdivided into psychrophilic, mesophilic, and thermophilic bacteria. In earlier studies, constant temperatures were used to incubate and isolate bacterial strains, such as at low temperature (Song et al., 2009), extreme high temperature (Gonzalez et al., 1996), and mesophilic temperature (Sun et al., 2014b); very few studies used ambient-temperatures. The present study suggested that using ambient-temperature during the enrichment step could significantly enhance bacterial culturability. This may be attributed to the circadian rhythm. Previous studies have demonstrated that prokaryotes also follow circadian rhythms, similar to those experience by eukaryotes (Huang et al., 1990). Furthermore, this rhythm is temperature-dependent (Sweeney and Borgese, 1989). The natural environment shows oscillating temperatures that are correlated with day/night cycle, such as the temperature of the top layer of soils. Because of this endogenous “circadian clocks,” indigenous microbes may have adapted to oscillating temperatures along with day/night cycles, thus forming the special bacterial circadian rhythm found in the natural environment. As a result, incubation at constant temperature may disturb the inherent rhythm of bacterial strains in the environment, which leads to reduced culturability. Additionally, each strain has an inherent optimal temperature for its metabolism. A constant incubation temperature may only be able to provide the optimal temperature for a small number of the strains, while the other strains are kept in the dormant phase or grow at much slower rates due to the unchanging temperature. Therefore, ambient temperatures that change with time may provide a wider growth window for more bacterial strains; however, they may grow slowly under this condition. The above two reasons may explain why using ambient temperatures could enhance culturability of strains isolated from the environment.

In earlier studies, bacterial strains harboring the *alkB*/*alkM* gene were found to affected by the isolation medium (Sun et al., 2014b). In the present study, isolation of bacteria containing various phenol and alkane hydroxylase genes was also affected by the isolating condition, namely the cultivating mode and temperature. The different kinds of alkane or phenol hydroxylase genes may bestow the host with different alkane- or phenol-degrading abilities (Nie et al., 2014b; Sun et al., 2014a,b). In fact, enrichment using specific carbon sources is a selective process of survival of the fittest, during which a large number of strains without alkane-degrading abilities or with inefficient alkane-degrading abilities are eliminated. The different alkane-hydroxylase catalysis ability could ultimately determine the survival of bacterial strains. More investigations need to be made on the mechanisms that underlie the relationship between the temperature and bacterial genes clusters.

Results from the present study also suggested that the direct-plating method could result in higher percentage of hexadecane- and phenol-degrading isolates as compared with that obtained via enrichment using mineral salt medium and oil-production water. The lower number of alkane- or phenol-degrading isolates in the enrichment method may be due to the abundance of rare microorganism that could not degrade crude oil. In this system, metabolites from crude oil degraders provided other bacteria with essential nutrients. In addition, crude oil contains diverse carbon sources, which can be easily used by many bacterial strains for growth. Because of the existence of the easily degradable carbon source, strains that are unable to degrade AHs also could grow vigorously in the enrichment culture. Therefore, the process of enrichment with crude oil may decline the percentage of n-hexadecane degraders.

It has been shown that *pheN, CYP153A, alkB*, and *almA* genes correlated with the phylogeny of their host. For example, *CYP153A, alkB*, and *almA* genes from *Marinobacter* strains were often tightly clustered (Figures 3–5). In contrast, many diverse strains also contained near similar genes. For example, the *alkB* genes from *Acinetobacter* sp. SLG310A2-9, *Rhizobium* sp. SLG510A10-12, and *Microbacterium* sp. SLG510B10-40B1-2 were found to be highly similar to those from *Acinetobacter venetianus* (AEW67043) (Mara et al., 2012). This may be attributed to horizontal gene transfer (HGT), which has been shown to be ubiquitous in the environment (de la Cruz and Davies, 2000). More interestingly, the less-oil-contaminated soil (S1) also possessed numerous alkane-degrading bacterial strains; some bacterial strains exhibited three different types of alkane-degrading genes. This suggested that alkane-degrading strains are ubiquitously distributed in the natural environments regardless of oil contamination.

It is worth noting that the occurrence of the multiple *alkB, CYP153A*, and *almA* alkane hydroxylases in one bacterium is quite frequent in *Marinobacter* strains. The coexistence of multiple alkane hydroxylases was also observed in *Dietzia* sp. DQ12-45-1b (Nie et al., 2011), *Amycolicicoccus subflavus* DQS3-9A1^T^ (AEF42720) (Nie et al., 2013), *Acinetobacter* sp. ADP1 (Barbe et al., 2004), and many *Alcanivorax* isolates (Wang et al., 2010; Wang and Shao, 2012). These three alkane hydroxylases demonstrated complementary substrate ranges: CYP153A usually catalyzed the hydroxylation of the alkane with C5-C10, AlkB catalyzed C5-C16, while AlmA catalyzed C32-C36. The coexistence of alkane hydroxylase genes undoubtedly broadened the substrate range, thereby enhancing the adaptive ability of the host. While many strains showed tremendous growth in MSM containing *n*-hexadecane as the sole carbon and energy source, none of three abovementioned genes were amplified from them, including *Rhizobium* sp. SL014B-37A2, *Stappia* sp. SL014B-87A3, *Labrenzia* sp. SL014B-4A2, *Paracoccus* sp. SL013B-8A1, *Planococcus* sp. SL013A-41A-2, *Pseudomonas* spp. SLG510A3-14, *Enterobacter* sp. SLG510A3-6, and *Bacillus* spp. SLG210A2-18-1 and SLG210A2-29A1. It is possible that these isolates harbored other alkane hydroxylases, which should be investigated in the future.

In summary, under different incubation conditions, 595 bacterial strains were isolated from oil contaminated soils. These strains were affiliated into 56 genera belonging to Actinobacteria, Proteobacteria, Firmicutes, and Bacteroidetes. Using the enrichment method, 239 isolates distributed into 31 genera, and 96 isolates distributed into 22 genera were obtained by culturing at ambient temperatures and a constant temperature, respectively, from a single soil sample. With the direct-plating method, 97 isolates affiliated with 15 genera and 163 isolates affiliated with 20 genera were obtained by culturing under ambient temperatures and a constant temperature, respectively, from two soil samples. Furthermore, phenol hydroxylase genes, short- and medium-chain alkane monooxygenase AlkB and CYP153A, and long-chain alkane monooxygenase AlmA were detected in numerous bacterial strains, including some genera for the first time. We found that several strains could utilize phenol and *n*-alkanes as the sole carbon source for growth, but no known related genes were found in these strains, suggesting the existence of novel genes in these strains that need to be further studied in the future.

## Author contributions

J-QS and X-LW designed the research. J-QS and LX performed the research. X-YL analyzed the 16S rRNA sequences. G-FZ and HC participated in isolation of bacterial strains. J-QS and YN wrote the paper.

### Conflict of interest statement

The authors declare that the research was conducted in the absence of any commercial or financial relationships that could be construed as a potential conflict of interest.
