# Supplementary material for: Functional Genetic Diversity and Culturability of Petroleum-Degrading Bacteria Isolated From Oil-Contaminated Soils
Source: Front Microbiol. 2018 Jun 20;9:1332. doi: 10.3389/fmicb.2018.01332 (PMC6019457; doi:10.3389/fmicb.2018.01332)
Supplement: Supplementary file 1 [file Table_1.DOCX]

***Supplemental information***

Functional genetic diversity and culturability of petroleum-degrading bacteria isolated from oil-contaminated soils

Ji-Quan Sun^1^*, Lian Xu^1^*, Xue-Ying Liu^1^, Gui-Fang Zhao^2^, Hua Cai^2^, Yong Nie^1^, Xiao-Lei Wu^1#^

1. College of Engineering, Peking University, Beijing 100871, PR China

2. School of Environment, Tsinghua University, Beijing 100084, PR China

***Authors have contributed equally to this work**

**# Corresponding author:**

**Xiao-Lei Wu,** College of Engineering, Peking University, Beijing 100871, People’s Republic of China, Tel/Fax: +86-10-62759047. Email: xiaolei_wu@pku.edu.cn

**Table S1 Characteristics of the soils which used for the isolation of strains**

| Characteristics | Control Soil (S0) | Oil-Contaminated soil (S1) |
| --- | --- | --- |
| Soil kind | Sandy loam soil | Sandy loam soil |
| Organic contents (g/kg) | 11.48 | 19.43 |
| Soluble salts (μS/cm) | 3800 | 1500 |
| Humic acid (%) | 0.13 | 0.11 |
| Water-soluble nitrogen (mg/kg) | 20.90 | 50.56 |
| Available phosphorus (mg/kg) | 11.52 | 11.51 |
| Total nitrogen (%) | 0.015 | 0.028 |
| Total Phosphorus (%) | 0.074 | 0.070 |
| K^+^ (%) | 1.89 | 1.72 |
| Ca (%) | 5.16 | 5.04 |
| Na (%) | 1.41 | 1.39 |
| Mg (%) | 1.25 | 1.16 |
| Fe (%) | 2.47 | 2.35 |
| Mn (%) | 0.045 | 0.039 |
| Cu (mg/kg) | 5.0 | 7.00 |
| Zn (mg/kg) | 42.0 | 34.00 |
| Si (%) | 28.98 | 29.58 |
