# Supplementary material for: Functional Genetic Diversity and Culturability of Petroleum-Degrading Bacteria Isolated From Oil-Contaminated Soils
Source: Front Microbiol. 2018 Jun 20;9:1332. doi: 10.3389/fmicb.2018.01332 (PMC6019457; doi:10.3389/fmicb.2018.01332)
Supplement: Supplementary file 2 [file Table_2.DOCX]

***Supplemental information***

Functional genetic diversity and culturability of petroleum-degrading bacteria isolated from oil-contaminated soils

Ji-Quan Sun^1^*, Lian Xu^1^*, Xue-Ying Liu^1^, Gui-Fang Zhao^2^, Hua Cai^2^, Yong Nie^1^, Xiao-Lei Wu^1#^

1. College of Engineering, Peking University, Beijing 100871, PR China

2. School of Environment, Tsinghua University, Beijing 100084, PR China

***Authors have contributed equally to this work**

**# Corresponding author:**

**Xiao-Lei Wu,** College of Engineering, Peking University, Beijing 100871, People’s Republic of China, Tel/Fax: +86-10-62759047. Email: xiaolei_wu@pku.edu.cn

**Table S2 The different isolating condition of the strains**

|  | Isolation source | Temperature | Enrichment | Pyruvate | Medium | Carbon source |
| --- | --- | --- | --- | --- | --- | --- |
| SL013A | S1 | Ambient | No | N | PW | Oil production water |
| SL013B | S1 | 30 | No | N | PW | Oil production water |
| SL014A | S1 | Ambient | No | N | PM | Yeast extract |
| SL014B | S1 | 30 | No | N | PM | Yeast extract |
| SL003A | S0 | Ambient | No | N | PW | Oil production water |
| SL003B | S0 | 30 | No | N | PW | Oil production water |
| SL004A | S0 | Ambient | No | N | PM | Yeast extract |
| SL004B | S0 | 30 | No | N | PM | Yeast extract |
| SLG210A2 | S0 | Ambient | Yes | N | G2 | Crude Oil/Yeast extract |
| SLG210A3 | S0 | Ambient | Yes | N | G2 | Crude Oil/Yeast extract |
| SLG210B1 | S0 | 30 | Yes | N | G2 | Crude Oil/Yeast extract |
| SLG310A2 | S0 | Ambient | Yes | N | G3 | Crude Oil |
| SLG310B1 | S0 | 30 | Yes | N | G3 | Crude Oil |
| SLG310B2 | S0 | 30 | Yes | N | G3 | Crude Oil |
| SLG510A3 | S0 | Ambient | Yes | N | G5 | Crude Oil |
| SLG510A10 | S0 | Ambient | Yes | N | G5 | Crude Oil |
| SLG510B2 | S0 | 30 | Yes | N | G5 | Crude Oil |
| SLG510B10 | S0 | 30 | Yes | N | G5 | Crude Oil |
