# Supplementary material for: Functional Genetic Diversity and Culturability of Petroleum-Degrading Bacteria Isolated From Oil-Contaminated Soils
Source: Front Microbiol. 2018 Jun 20;9:1332. doi: 10.3389/fmicb.2018.01332 (PMC6019457; doi:10.3389/fmicb.2018.01332)

Supplemental information

Functional genetic diversity and culturability of petroleum-degrading bacteria isolated from oil-contaminated soils

Ji-Quan Sun[1]*, Lian Xu[1]*, Xue-Ying Liu[1], Gui-Fang Zhao[2], Hua Cai[2], Yong Nie[1], Xiao-Lei Wu[1#]

1. College of Engineering, Peking University, Beijing 100871, PR China

2. School of Environment, Tsinghua University, Beijing 100084, PR China

***Authors have contributed equally to this work**

# Corresponding author:

Xiao-Lei Wu, College of Engineering, Peking University, Beijing 100871, People's Republic of China, Tel/Fax: +86-10-62759047. Email: xiaolei_wu@pku.edu.cn

Fig. S1 Phylogenetic Neighbor-Joining tree of the 16S rRNA genes from the soils and bacterial isolates. The tree topology was evaluated by bootstrap analysis based on 1000 resampling replicates with MEGA 6.0. The bootstrap values (%) are indicated at the nodes (only greater than 50% were shown, the same below). The strains isolated by using ambient temperature were typed in bold with red color, the isolates by using constant 30°C were typed in bold with blue color, and the sequences from the clone library were typed in bold with green color. The numbers in square brackets are the numbers of the isolates within this phylotype.

Fig. S1

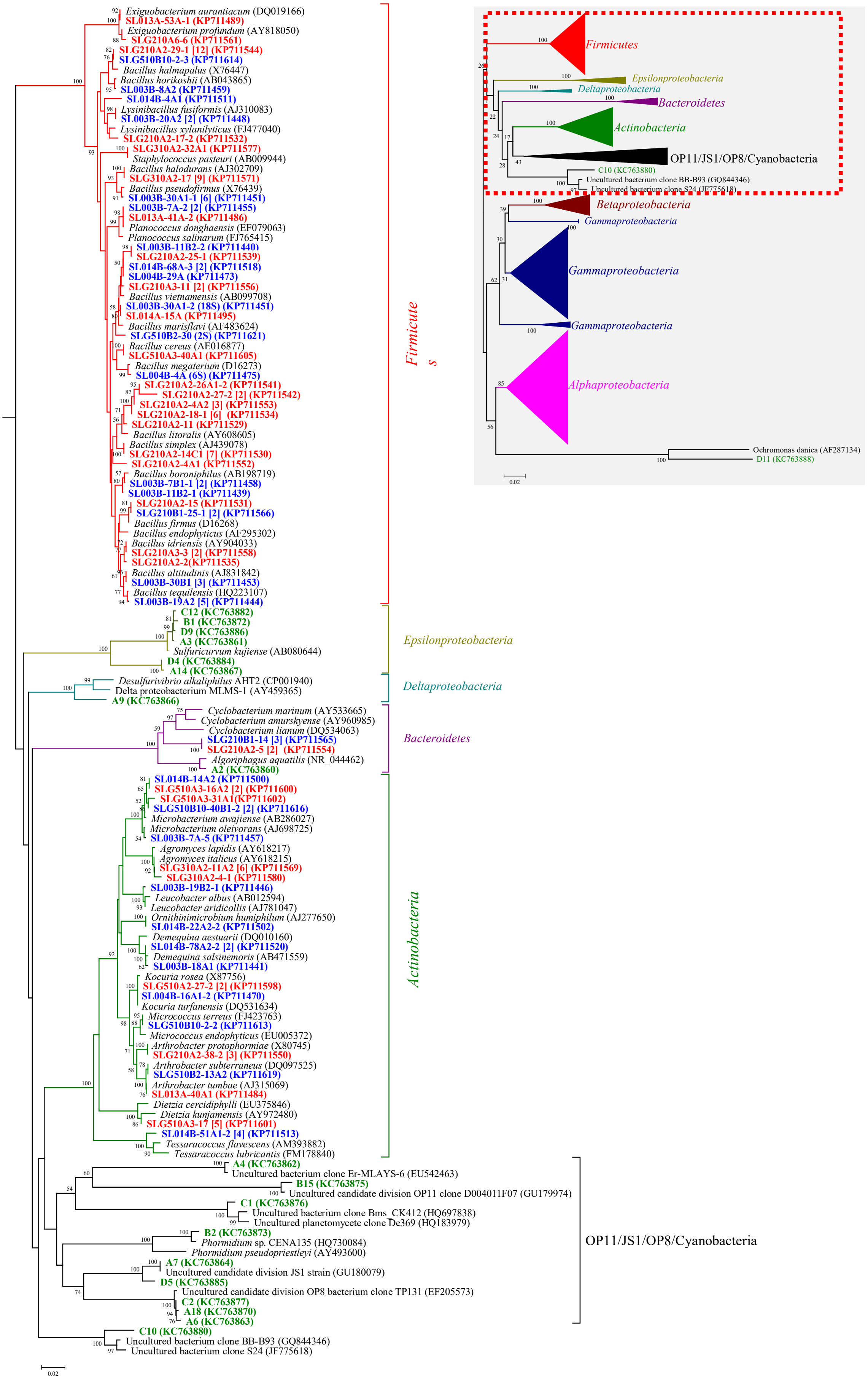

Exiguobacterium aurantiacum (DQ019166)
SL013A-53A-1 (KP711489)
Exiguobacterium profundum (AY818050)
SLG210A6-6 (KP711561)
SLG210A2-29-1 [12] (KP711544)
SLG510B10-2-3 (KP711614)
Bacillus halmapalus (X76447)
Bacillus horikoshii (AB043865)
SL003B-8A2 (KP711459)
SL014B-4A1 (KP711511)
Lysinibacillus fusiformis (AJ310083)
SL003B-20A2 [2] (KP711448)
Lysinibacillus xylanilyticus (FJ477040)
SLG210A2-17-2 (KP711532)
SLG310A2-32A1 (KP711577)
Staphylococcus pasteuri (AB009944)
Bacillus halodurans (AJ302709)
SLG310A2-17 [9] (KP711571)
Bacillus pseudofirmus (X76439)
SL003B-30A1-1 [6] (KP711451)
SL003B-7A-2 [2] (KP711455)
SL013A-41A-2 (KP711486)
Planococcus donghaensis (EF079063)
Planococcus salinarum (FJ765415)
SL003B-11B2-2 (KP711440)
SLG210A2-25-1 (KP711539)
SL014B-68A-3 [2] (KP711518)
SL004B-29A (KP711473)
SLG210A3-11 [2] (KP711556)
Bacillus vietnamensis (AB099708)
SL003B-30A1-2 (18S) (KP711451)
SL014A-15A (KP711495)
Bacillus marisflavi (AF483624)
SLG510B2-30 (2S) (KP711621)
Bacillus cereus (AE016877)
SLG510A3-40A1 (KP711605)
Bacillus megaterium (D16273)
SL004B-4A (6S) (KP711475)
SLG210A2-26A1-2 (KP711541)
SLG210A2-27-2 [2] (KP711542)
SLG210A2-4A2 [3] (KP711553)
SLG210A2-18-1 [6] (KP711534)
SLG210A2-11 (KP711529)
Bacillus litoralis (AY608605)
Bacillus simplex (AJ439078)
SLG210A2-14C1 [7] (KP711530)
SLG210A2-4A1 (KP711552)
Bacillus boroniphilus (AB198719)
SL003B-7B1-1 [2] (KP711458)
SL003B-11B2-1 (KP711439)
SLG210A2-15 (KP711531)
SLG210B1-25-1 [2] (KP711566)
Bacillus firmus (D16268)
Bacillus endophyticus (AF295302)
Bacillus idriensis (AY904033)
SLG210A3-3 [2] (KP711558)
SLG210A2-2(KP711535)
Bacillus altitudinis (AJ831842)
SL003B-30B1 [3] (KP711453)
Bacillus tequilensis (HQ223107)
SL003B-19A2 [5] (KP711444)
Firmicutes
C12 (KC763882)
B1 (KC763872)
D9 (KC763886)
A3 (KC763861)
Sulfuricurvum kujiense (AB080644)
D4 (KC763884)
A14 (KC763867)
Epsilonproteobacteria
Desulfurivibrio alkaliphilus AHT2 (CP001940)
Delta proteobacterium MLMS-1 (AY459365)
A9 (KC763866)
Deltaproteobacteria
Cyclobacterium marinum (AY533665)
Cyclobacterium amurskyense (AY960985)
Cyclobacterium lianum (DQ534063)
SLG210B1-14 [3] (KP711565)
SLG210A2-5 [2] (KP711554)
Algoriphagus aquatilis (NR_044462)
A2 (KC763860)
Bacteroidetes
SL014B-14A2 (KP711500)
SLG510A3-16A2 [2] (KP711600)
SLG510A3-31A1(KP711602)
SLG510B10-40B1-2 [2] (KP711616)
Microbacterium awajiense (AB286027)
Microbacterium oleivorans (AJ698725)
SL003B-7A-5 (KP711457)
Agromyces lapidis (AY618217)
Agromyces italicus (AY618215)
SLG310A2-11A2 [6] (KP711569)
SLG310A2-4-1 (KP711580)
SL003B-19B2-1 (KP711446)
Leucobacter albus (AB012594)
Leucobacter aridicollis (AJ781047)
Ornithinimicrobium humiphilum (AJ277650)
SL014B-22A2-2 (KP711502)
Demequina aestuarii (DQ010160)
SL014B-78A2-2 [2] (KP711520)
Demequina salsinemoris (AB471559)
SL003B-18A1 (KP711441)
Kocuria rosea (X87756)
SLG510A2-27-2 [2] (KP711598)
SL004B-16A1-2 (KP711470)
Kocuria turfanensis (DQ531634)
Micrococcus terreus (FJ423763)
SLG510B10-2-2 (KP711613)
Micrococcus endophyticus (EU005372)
Arthrobacter protophormiae (X80745)
SLG210A2-38-2 [3] (KP711550)
Arthrobacter subterraneus (DQ097525)
SLG510B2-13A2 (KP711619)
Arthrobacter tumbae (AJ315069)
SL013A-40A1 (KP711484)
Dietzia cercidiphylli (EU375846)
Dietzia kunjamensis (AY972480)
SLG510A3-17 [5] (KP711601)
SL014B-51A1-2 [4] (KP711513)
Tessaracoccus flavescens (AM393882)
Tessaracoccus lubricantis (FM178840)
Actinobacteria
A4 (KC763862)
Uncultured bacterium clone Er-MLAYS-6 (EU542463)
B15 (KC763875)
Uncultured candidate division OP11 clone D004011F07 (GU179974)
C1 (KC763876)
Uncultured bacterium clone Bms_CK412 (HQ697838)
Uncultured planctomycete clone De369 (HQ183979)
B2 (KC763873)
Phormidium sp. CENA135 (HQ730084)
Phormidium pseudopriestleyi (AY493600)
A7 (KC763864)
Uncultured candidate division JS1 strain (GU180079)
D5 (KC763885)
Uncultured candidate division OP8 bacterium clone TP131 (EF205573)
C2 (KC763877)
A18 (KC763870)
A6 (KC763863)
OP11/JS1/OP8/Cyanobacteria
C10 (KC763880)
Uncultured bacterium clone BB-B93 (GQ844346)
Uncultured bacterium clone S24 (JF775618)
0.02
Firmicutes
Epsilonproteobacteria
Deltaproteobacteria
Bacteroidetes
Actinobacteria
OP11/JS1/OP8/Cyanobacteria
C10 (KC763880)
Uncultured bacterium clone BB-B93 (GQ844346)
Uncultured bacterium clone S24 (JF775618)
Betaproteobacteria
Gammaproteobacteria
Gammaproteobacteria
Gammaproteobacteria
Alphaproteobacteria
Ochromonas danica (AF287134)
D11 (KC763888)
0.02

Fig. S1 continue

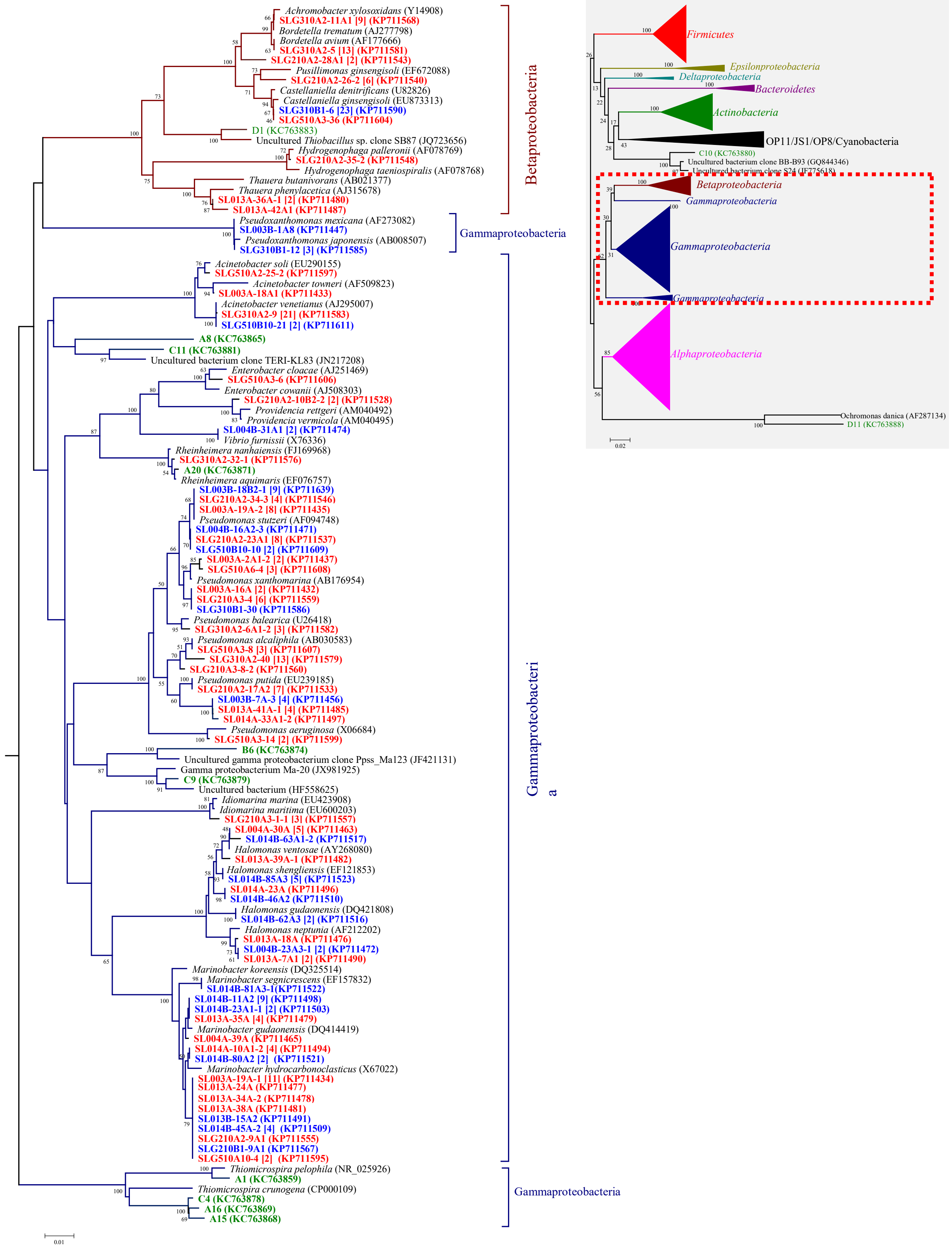

Fig. S1 continue

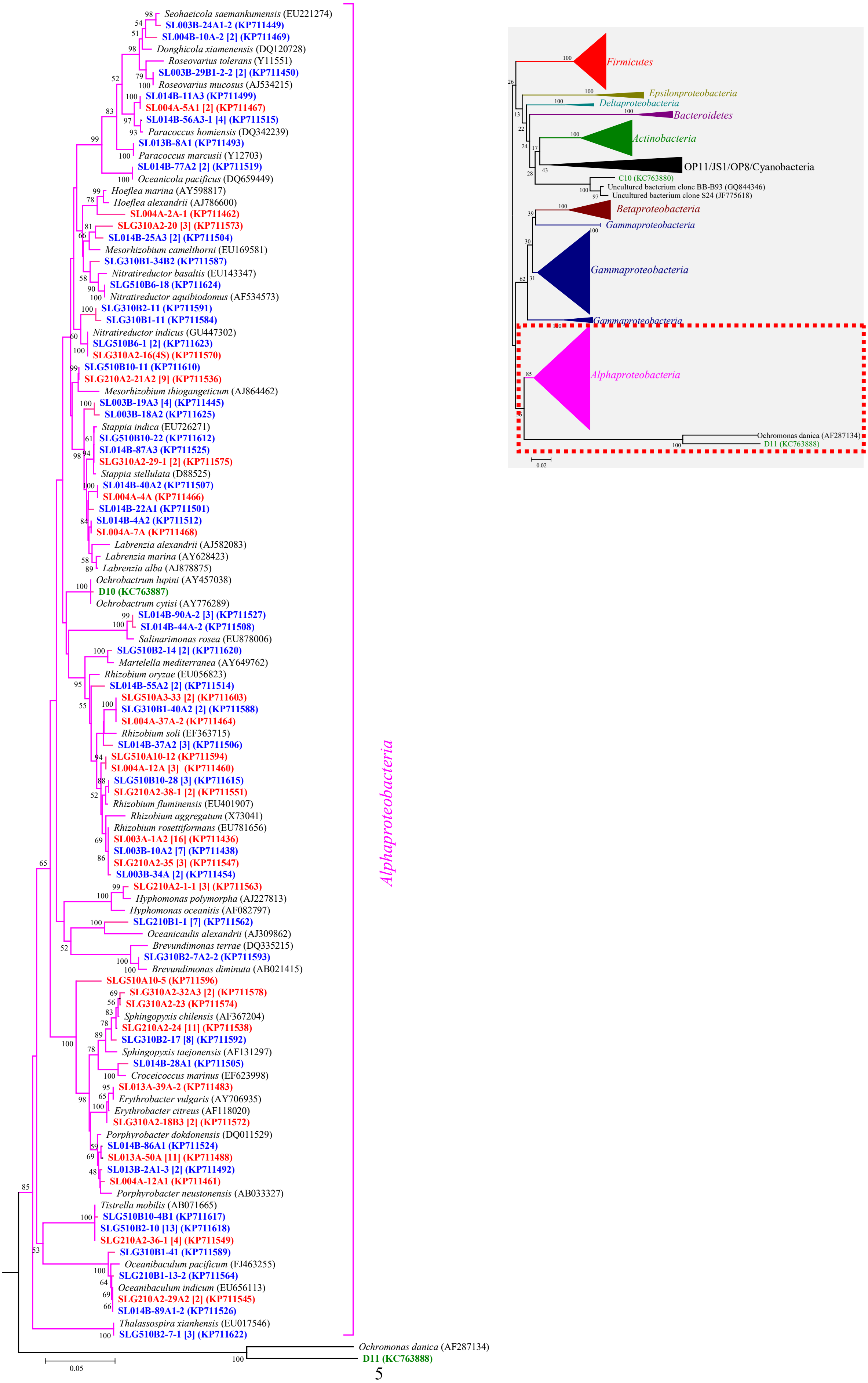

Supplement: Supplementary file 3 [file Image_1.PDF]
