# Supplementary material for: Functional Genetic Diversity and Culturability of Petroleum-Degrading Bacteria Isolated From Oil-Contaminated Soils
Source: Front Microbiol. 2018 Jun 20;9:1332. doi: 10.3389/fmicb.2018.01332 (PMC6019457; doi:10.3389/fmicb.2018.01332)

Supplemental information

Functional genetic diversity and culturability of petroleum-degrading bacteria isolated from oil-contaminated soils

Ji-Quan Sun[1]*, Lian Xu[1]*, Xue-Ying Liu[1], Gui-Fang Zhao[2], Hua Cai[2], Yong Nie[1], Xiao-Lei Wu[1#]

1. College of Engineering, Peking University, Beijing 100871, PR China

2. School of Environment, Tsinghua University, Beijing 100084, PR China

***Authors have contributed equally to this work**

# Corresponding author:

Xiao-Lei Wu, College of Engineering, Peking University, Beijing 100871, People's Republic of China, Tel/Fax: +86-10-62759047. Email: xiaolei_wu@pku.edu.cn

Fig.S2 Distribution of genus presenting on different media (a) and different temperature(b)

a

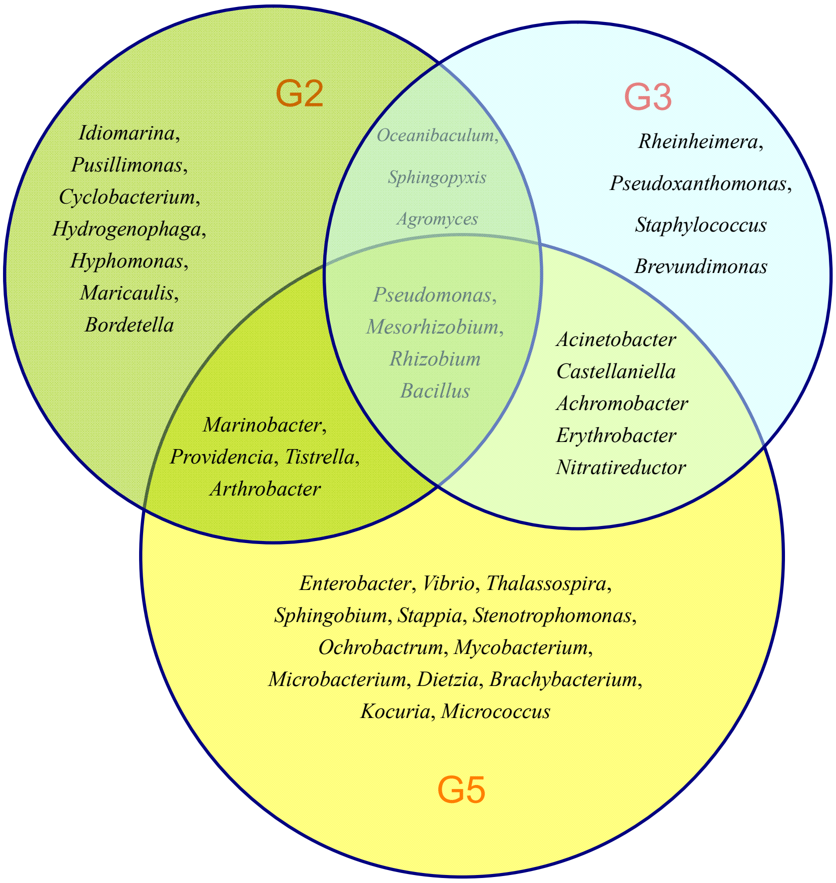

b

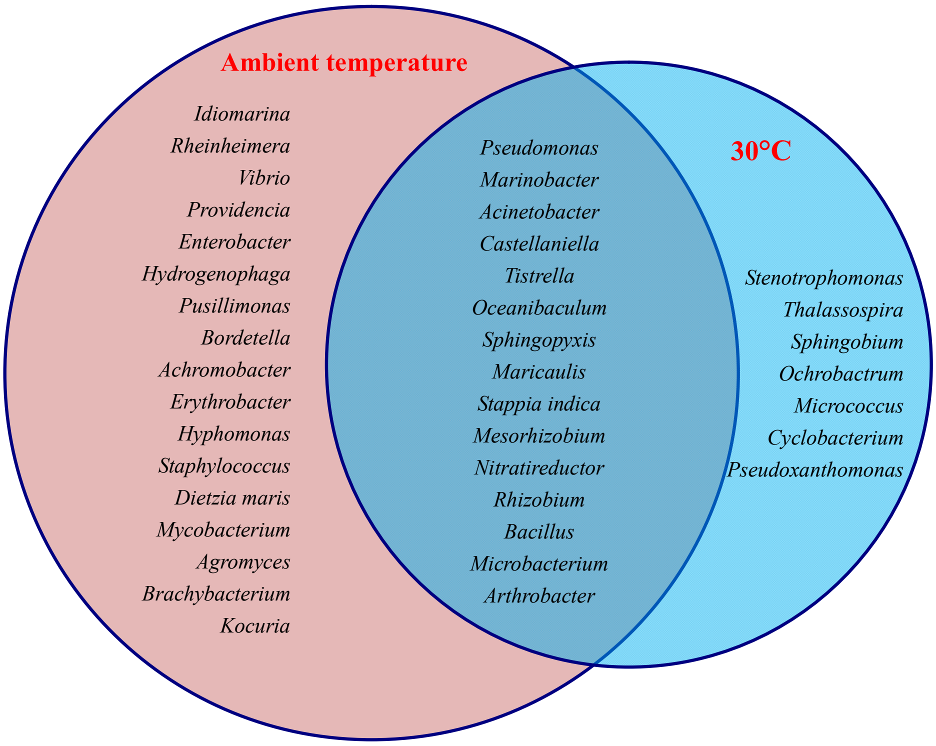

Supplement: Supplementary file 4 [file Image_2.PDF]
